# Biomimetic Hydrogel‐Mediated Mechano‐Immunometabolic Therapy for Inhibition of ccRCC Recurrence After Surgery

**DOI:** 10.1002/advs.202308734

**Published:** 2024-06-17

**Authors:** Yunze Dong, Jun Luo, Mingliang Pei, Shuai Liu, Yuchen Gao, Hongmin Zhou, Yimingniyizi Nueraihemaiti, Xiangcheng Zhan, Tiancheng Xie, Xudong Yao, Xin Guan, Yunfei Xu

**Affiliations:** ^1^ Department of Urology Shanghai Tenth People's Hospital School of Medicine Tongji University Shanghai 200072 P. R. China; ^2^ Department of Urology Shanghai Fourth People's Hospital School of Medicine Tongji University Shanghai 200434 P. R. China; ^3^ Department of Orthopaedics Shanghai Key Laboratory for Prevention and Treatment of Bone and Joint Diseases Shanghai Institute of Traumatology and Orthopaedics Ruijin Hospital Shanghai Jiao Tong University School of Medicine Shanghai 200025 P. R. China; ^4^ Department of Ultrasound Institute of Ultrasound in Medicine and Engineering Zhongshan Hospital Fudan University Shanghai 200032 P. R. China

**Keywords:** clear cell renal cell carcinoma, immunometabolic therapy, indoleamine 2,3‐dioxygenase 1, tumor physical microenvironment, tumor recurrence

## Abstract

The unique physical tumor microenvironment (TME) and aberrant immune metabolic status are two obstacles that must be overcome in cancer immunotherapy to improve clinical outcomes. Here, an in situ mechano‐immunometabolic therapy involving the injection of a biomimetic hydrogel is presented with sequential release of the anti‐fibrotic agent pirfenidone, which softens the stiff extracellular matrix, and small interfering RNA IDO1, which disrupts kynurenine‐mediated immunosuppressive metabolic pathways, together with the multi‐kinase inhibitor sorafenib, which induces immunogenic cell death. This combination synergistically augmented tumor immunogenicity and induced anti‐tumor immunity. In mouse models of clear cell renal cell carcinoma, a single‐dose peritumoral injection of a biomimetic hydrogel facilitated the perioperative TME toward a more immunostimulatory landscape, which prevented tumor relapse post‐surgery and prolonged mouse survival. Additionally, the systemic anti‐tumor surveillance effect induced by local treatment decreased lung metastasis by inhibiting epithelial‐mesenchymal transition conversion. The versatile localized mechano‐immunometabolic therapy can serve as a universal strategy for conferring efficient tumoricidal immunity in “cold” tumor postoperative interventions.

## Graphical abstract

Postoperative recurrence of clear cell renal cell carcinoma is associated with a poor clinical outcome. Herein, this study designs a biomimetic hydrogel system to reprogram tumor immune microenvironment from an immune‐excluded to the immune‐inflamed landscape via mechanically remodeling the tumor physical microenvironment and blocking IDO1/Kyn/AhR pathways, showing reduced post‐surgical cancer recurrence and metastasis.

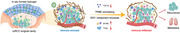

## 1 Introduction

Clear cell renal cell carcinoma (ccRCC) is a common malignancy of the urinary system that poses a serious threat to human health.^[^ ^1^, ^2^ ^]^ Surgery is the first‐line treatment for ccRCC, however, a poor response to chemotherapy and radiotherapy and a high recurrence rate of 40% after surgical resection remain critical clinical issues that urgently need to be addressed.^[^ ^3^, ^4^, ^5^ ^]^ Among the various factors that result in tumor insensitivity or relapse, the unique stiff and fibrotic stroma and altered cellular metabolism in the tumor microenvironment (TME) that favors tumor cell survival but adversely affects tumor‐infiltrating immune cells have been extensively investigated.^[^ ^6^, ^7^, ^8^ ^]^

As a pivotal trait of the TME, the dense and rigid extracellular matrix (ECM) in solid tumors reduces tumor perfusion by compressing blood vessels, leading to restricted drug enrichment, oxygen deficiency, and finite trafficking of immune cells.^[^ ^9^, ^10^ ^]^ In particular, excessive collagen synthesis and deposition are the pathological processes responsible for increased ECM stiffness, mainly involving activated cancer‐associated fibroblasts (CAFs).^[^ ^6^, ^11^, ^12^, ^13^ ^]^ The activated CAFs can build and remodel ECM structures, which not only form a physical barrier to impede therapeutic agent delivery and cytotoxic T lymphocyte (CTL) infiltration but also secrete multiple pro‐tumorigenic cytokines such as transforming growth factor‐β1 (TGF‐β1), interleukin‐6 (IL‐6), and CC‐chemokine ligand 2 to abrogate immune responses to support tumor immune evasion.^[^ ^14^, ^15^, ^16^ ^]^ Bioinformatics analysis based on The Cancer Genome Atlas (TCGA) database of ccRCC patients showed that TGF‐β1 was upregulated, which was negatively associated with patient overall survival (Figure S1, Supporting Information), implying that mechanical intervention of ECM components may be conducive to CTL infiltration and activation.

The overexpression of immunosuppressive enzymes within the TME also exerts a crucial influence on tumor progression and immunosuppression.^[^ ^17^, ^18^, ^19^ ^]^ Tumor cells compete with immune cells, especially T lymphocytes, for amino acids and other nutrients to fuel their progression.^[^ ^8^ ^]^ This has spurred the identification of diverse metabolites that regulate immune cell function through different pathways. Among these metabolites, tryptophan (Trp) and its catabolite kynurenine (Kyn), which are produced via the indoleamine 2,3‐dioxygenase (IDO1)/Trp 2,3‐dioxygenase pathway, were carefully studied.^[^ ^20^, ^21^, ^22^, ^23^ ^]^ Theoretically, Kyn production and Trp consumption contribute to immunosuppression.^[^ ^24^ ^]^ The local reduction of Trp concentration owing to its catabolism activates general control nonderepressible 2 kinase and inhibits the mechanistic target of the rapamycin pathway, whereas increased local Kyn concentration stimulates the aryl hydrocarbon receptor (AhR) that facilitates differentiation of regulatory cells (Tregs) and M2‐like tumor‐associated macrophages (M2‐TAMs).^[^ ^24^, ^25^, ^26^ ^]^ Bioinformatics and western blotting analyses of ccRCC patients and normal controls revealed that IDO1 was significantly more highly expressed in the renal tissues of ccRCC patients (523 cases) than in those of health controls (100 cases) (Figure S2A,C, Supporting Information). Survival stratification showed that ccRCC patients with lower IDO1 expression tended to have an extended overall survival. Given that these metabolites act as checkpoints, targeting them could offer an alternative avenue for reinvigorating anti‐tumor immunity.

In addition to immunonegative factors, sufficient immunogenicity is required to initiate the T cell immune cycle for a potent immune response.^[^ ^27^ ^]^ Among multiple targeted drugs that are utilized in clinical ccRCC treatment, sorafenib (Sfn), a multi‐targeted tyrosine kinase inhibitor, can directly or indirectly promote tumor immunogenicity by blocking RAF/MEK/ERK‐mediated cell signaling pathways or by inhibiting VEGFR/PDGF receptors.^[^ ^28^, ^29^, ^30^ ^]^ Pirfenidone (PFD), a promising anti‐fibrotic drug, has been approved by the U.S. Food and Drug Administration (FDA) for the clinical treatment of idiopathic pulmonary fibrosis.^[^ ^31^, ^32^ ^]^ Evidence suggests that PFD inhibits TGF‐β1‐induced myofibroblast differentiation and collagen synthesis, thereby remodeling the ECM.^[^ ^33^ ^]^ In addition, small interfering RNA targeting IDO1 (siIDO1) may relieve the immune brakes related to immunosuppressive cells, thus boosting peripheral T cell activation.^[^ ^34^, ^35^ ^]^ This provides a promising strategy for the rational combination of PFD, Sfn, and siIDO1 as an efficient mechano‐immunometabolic therapeutic regimen, softening the stiff tumor physical microenvironment (TPME) for T cell infiltration, strengthening tumor immunogenicity for T cell initiation, and remodeling metabolism for T cell activation. However, small‐molecule inhibitors or siRNAs are prone to rapid clearance during blood circulation, induction of resistance mutations, and off‐target binding toxicity.^[^ ^36^, ^37^ ^]^ Notably, systemic administration of several agents may result in additional superimposed side effects. Critical issues exist in achieving the targeted delivery of the above agents with sequential release and ensuring payload activity in one system. Considering that surgical debulking is the primary clinical intervention for ccRCC patients, in situ implantation of a hydrogel to concentrate immunostimulatory molecules within the resection cavity may provide an effective drug delivery approach.^[^ ^38^, ^39^ ^]^

**Figure 1:**
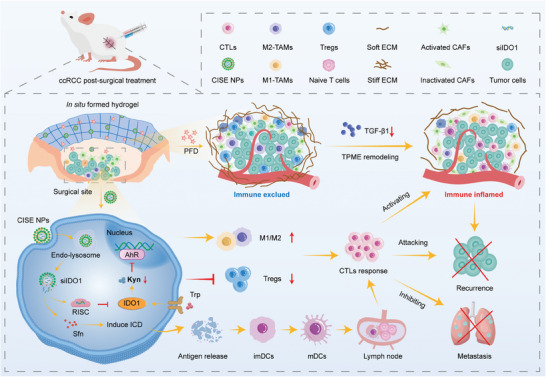
Schematic of the in situ biomimetic hydrogel system for postoperative ccRCC mechano‐immunometabolic therapy. Upon localized injection, the precursor solution gelatinizes rapidly within the resection site and sequential releasing anti‐fibrotic agent pirfenidone that softens stiff extracellular matrix and small interfering RNA IDO1 that disrupts IDO1/Kyn/AhR axis‐mediated immunosuppressive metabolic pathways, together with multi‐kinase inhibitor sorafenib that induces immunogenic cell death. This combination synergically facilitates the perioperative TME toward an immune inflamed landscape, which prevents ccRCC relapse post‐surgery and inhibits lung metastasis. CTLs, cytotoxic T lymphocytes; M1‐TAMs, M1‐like macrophages; M2‐TAMs, M2‐like macrophages; Tregs, regulatory T cells; mDC, mature dendritic cells; ECM, extracellular matrix; PFD, pirfenidone; TPME, tumor physical microenvironment; ICD, immunogenic cell death; CAFs, cancer‐associated fibroblasts.

Here, we developed a fibrinogen‐based biomimetic hydrogel as a drug reservoir for the co‐delivery of the anti‐fibrotic agents PFD and Cap‐siIDO1‐Sfn‐CD62E (CISE) NPs to create an immune‐stimulated niche to attack residual tumor cells and suppress ccRCC recurrence after surgery (**Figure** **1**). After localized injection, the precursor solution gelatinized rapidly within the resection site and the cargo was liberated in a sustained manner. First, PFD mechanically remodeled the ECM by reducing TGF‐β1 secretion and collagen deposition. The softened “physical barrier” then facilitated the liberalized CISE NPs to track and actively target residual tumor cells. Next, the internalized calcium phosphate nanocarriers disassembled upon endosomal acidic stimulation, and the payloads, including Sfn and siIDO1, were emitted from the endosomes to exert an immunomodulatory effect, which in turn mobilized CTLs and relieved Treg/M2‐TAMs‐related immune brakes to form an inflamed immune landscape for the prevention of postoperative ccRCC recurrence and metastasis.

## 2 Results

### 2.1 Synthesis and Characterizations of CISE‐PFD@Gel Hydrogel System

**Figure 2:**
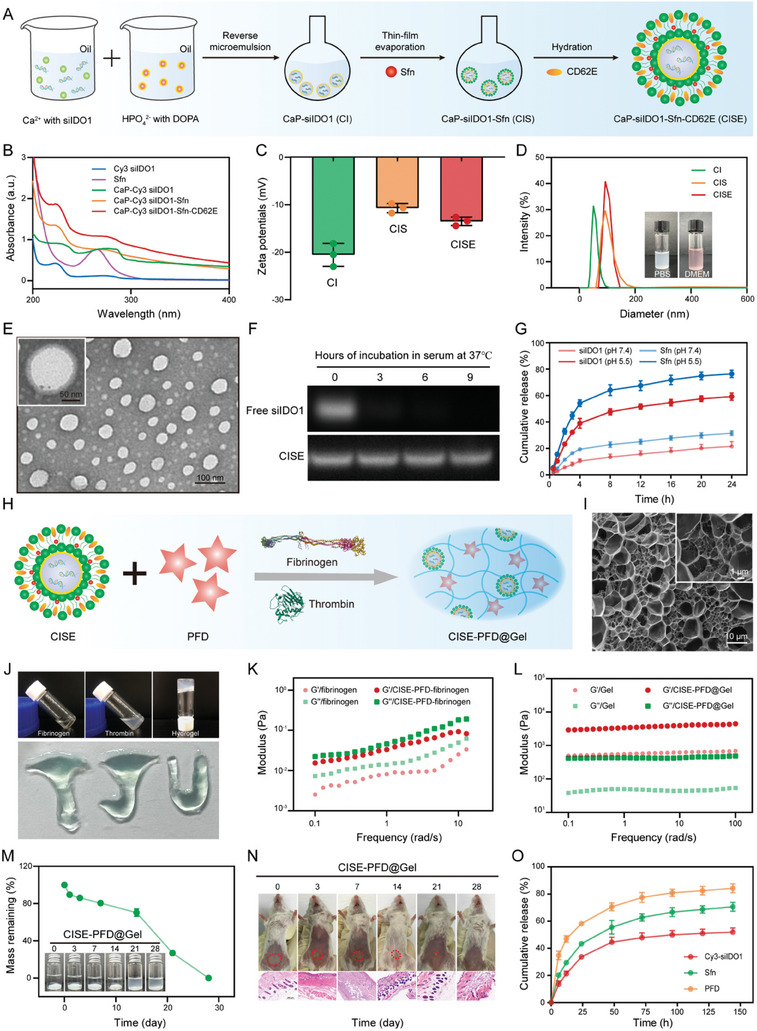
Synthesis and characterization of the biomimetic hydrogel system. A) Schematic illustration of the step‐by‐step synthesis of CISE NPs. B) UV–vis absorption curves of varied samples. C) Zeta potential variations in the preparation procedures of CISE NPs. D) Particle size distributions of CI, CIS, and CISE NPs measured by dynamic light scattering. Inset: digital photos of CISE NPs dispersed in PBS and cell culture medium. E) Representative TEM images of CISE NPs. F) Electrophoresis to evaluate the serum stability of free siIDO1 and siIDO1 reconstituted from CISE NPs. G) Cumulative release kinetics of Sfn and siIDO1 from CISE NPs in PBS (pH 7.4) or PBS (pH 5.5). H) Schematic of the drug‐loading hydrogel system preparation. I) Representative Cryo‐SEM images of the CISE‐PFD@Gel system. J) Representative digital photos of the hydrogel and the formation of diverse geometric shapes after syringe injection. K) Frequency spectra of the elastic (G′) and viscous (G″) moduli of CISE‐PFD‐fibrinogen precursor solution with the exhibiting solution‐like characteristics. L) Frequency spectra of the G′ and G″ moduli of CISE‐PFD@Gel system showing a gel‐like behavior. M) In vitro degradation profiles of CISE‐PFD@Gel system immersed in PBS over 28 days. N) In vivo degradation behavior and biocompatibility of the CISE‐PFD@Gel system. O) Cumulative release profiles of payload from CISE‐PFD@Gel system after intracavity implantation in postoperative mice model. Data were presented as means ± SD (n = 3).

CISE NPs were synthesized as shown in **Figure** **2**A. Three siRNA plasmids targeting IDO1 were designed and synthesized. Among these, siRNA‐3 was selected for further experiments owing to its ability to silence IDO1 mRNA expression (Figure S3, Supporting Information). Subsequently, pH‐responsive biodegradable CaP was used to condense the selected siIDO1 using the classical water‐in‐oil microemulsion approach with dioleoylphosphatidic acid (DOPA) electrostatically capped on the inner core surface. The prepared CaP‐siIDO1 (CI) core was mixed with an outer lipid layer composed of 1,2‐dimyristoyl‐sn glycerol‐3‐phosphocholine (DMPC), cholesterol, DSPE‐PEG‐2000, and Sfn to obtain Cap‐siIDO1‐Sfn (CIS) NPs. To achieve active tumor‐targeted delivery, CD62E protein was anchored to the CIS surface to yield the final CISE NPs. To visualize siIDO1, a Cy3‐labeled siRNA was synthesized as an alternative to siIDO1 for in vitro characterization. The UV–vis spectrum showed that Cy3 siRNA and Sfn were successfully encapsulated into Cap‐lipid nanocarriers, as evidenced by the characteristic absorption peaks at approximately 230 and 280 nm, respectively (Figure 2B). SDS‐PAGE showed that CISE NPs shared the same bands as the free CD62E protein, whereas CIS NPs did not, indicating efficient capping of CD62E onto CISE NPs (Figure S4, Supporting Information). The shifting zeta potential during stepwise synthesis also validated the successful construction of CISE NPs (Figure 2C). Dynamic light scattering measurements showed that the average particle size of the synthesized CISE NPs was ≈120 nm, with favorable dispersion and stability in phosphate‐buffered saline (PBS) and Dulbecco's modified Eagle's medium (Figure 2D; Figure S5, Supporting Information). Transmission electron microscopy images showed that the CISE NPs had a uniformly distributed spherical structure that was slightly smaller than the hydrated particle size, possibly due to solvation effects (Figure 2E). Meanwhile, siIDO1 reconstituted from CISE NPs remained stable after incubation with serum (10% vol) for 9 h, whereas free siIDO1 was entirely degraded during the same period (Figure 2F). An acidic TME can trigger degradation of the CaP core, resulting in the release of siIDO1 and Sfn. As shown in Figure 2G, CISE NPs exhibited faster drug release kinetics in an acidic environment (pH = 5.5) than in PBS at pH 7.4 for both payloads of siIDO1 and Sfn.

To locally deliver therapeutics at the resection site, we synthesized a biomimetic hydrogel system by mixing thrombin and fibrinogen solutions at a volume ratio of 1:2, simultaneously loaded with CISE NPs and the anti‐fibrotic drug PFD (Figure 2H). Cryo‐scanning electron microscopy revealed a porous and loose network structure in the gel system, which facilitated adequate drug loading (Figure 2I). Digital photographs revealed that thrombin catalyzes fibrinogen into a network that mimicks coagulation within seconds and can be smoothly injected to form diverse geometric shapes (Figure 2J). The solution‐gel conversion process was examined by rheological analysis. The frequency spectra of the fibrinogen/CISE‐PFD‐fibrinogen precursor solution displayed solution‐like properties, as evidenced by the fact that viscous moduli (G″) are greater than elastic moduli (G′) (Figure 2K). In contrast, both G′ and G″ levels increased with time in CISE‐PFD@Gel system, and the G′ level was remarkably higher than the G″ level, indicating successful gel formation (Figure 2L). The addition of CISE NPs and PFD retarded the gelation process but improved the elasticity of fibers. Meanwhile, the moduli of both the empty gel and CISE‐PFD@Gel system were independent of time, indicating that the biomimetic hydrogel system maintained high elasticity post‐injection (Figure S6, Supporting Information). Given that gel degradation is a prerequisite for payload release and action, we further evaluated the degradation behavior of the CISE‐PFD@Gel system under physiological conditions, both in vitro and in vivo. The gels placed in the PBS solution were almost completely degraded within approximately three weeks (Figure 2M). A similar degradation behavior was verified by injecting CISE‐PFD@Gel into the abdomen of mice (Figure 2N). More importantly, H&E staining of mouse abdominal skin tissues isolated at various time points after gel injection did not reveal any inflammatory cell deposition, demonstrating the biosafety of the as‐prepared CISE‐PFD@Gel system. As expected, all loadings demonstrated time‐dependent, sustained release from the hydrogel (Figure 2O). Within 72 h, 51.96% of siIDO1, 70.65% of Sfn, and 84.27% of PFD were released from the CISE‐PFD@Gel system. Notably, PFD exhibited faster release kinetics from the CISE‐PFD@Gel system compared with Sfn and siIDO1, possibly because of the protective effect of the CaP nanocarrier.

### 2.2 Cellular Uptake and Immunostimulation of the CISE NPs

**Figure 3:**
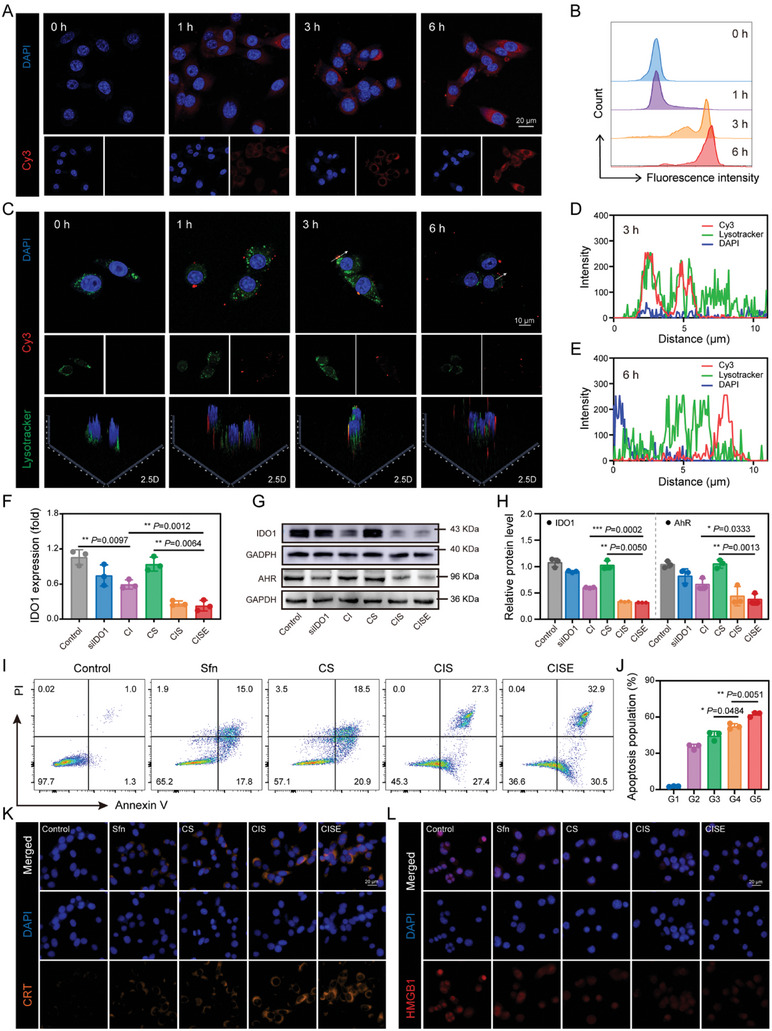
Cellular uptake and immunostimulation of CISE NPs. A) Representative CLSM images and B) relative quantification of fluorescently labeled CISE NPs uptake by Renca cells after varied times co‐incubation. C) Fluorescent visualization and of Cy3‐siRNA localization in Renca cells 0, 1, 3, 6 h after incubation with fluorescently labeled CISE NPs (Cy3‐siRNA, red; nuclei, blue and endosomes, green). D) and E) Corresponding mean fluorescence signal intensity of Renca cells cultured with Cy3‐labeled CISE NPs for 3 or 6 h. F) Gene‐silencing efficiency of CISE NPs determined via RT‐qPCR. G) Western blotting and H) relative quantification of IDO1 and AhR expression in Renca cells with various treatments. I) Representative flow cytometry images and J) relative quantification of apoptosis cells after various treatments. K) Representative immunofluorescence images of CRT and L) HMGB1 expressions in Renca cells after various treatments. Data were presented as means ± SD (n = 3). Statistical differences were calculated using a two‐tailed unpaired Student's *t*‐test. *P < 0.05, **P < 0.01, ***P < 0.001.

Endocytosis is one of the main obstacles to overcome during gene transfection.^[^ ^40^ ^]^ Cy3‐labeled siRNA was used instead of siIDO1 to determine the cellular uptake efficiency of CISE NPs using confocal laser scanning microscopy (CLSM) and flow cytometry (FCM). A gradual increase in the red fluorescence of CISE NPs (Cy3 siRNA instead of siIDO1) was observed in Renca cells with prolonged co‐incubation time (**Figure** **3**A). Quantitative analysis of intracellular fluorescence intensity by FCM showed that CISE NPs were effectively phagocytosed by Renca cells (Figure 3B). Following cell entry, the successful escape of nanoparticles from endo/lysosomes is critical for efficient gene transfection. Next, the endo/lysosomal escape capacity of CISE NPs was evaluated using CLSM in Renca cells. The red fluorescence of the Cy3‐labeled siRNA overlapped with the fluorescence of LysoTracker Green 3 h after incubation, indicating that CISE NPs were trapped inside lysosomes after endocytosis (Figure 3C,D). With prolonged incubation, Cy3 siRNA escaped from the lysosome and diffused into the cytoplasm, suggesting that internalized CISE NPs could disassemble under acidic stimulation within endosomes, and the payloads containing Cy3 siRNA and Sfn, were burst‐liberated from the endosomes (Figure 3C,E). After verifying the cytosolic delivery of the siRNA payload by CISE NPs, the gene‐silencing efficiency of the nanoformulation was assessed. CI, CIS, and CISE NPs silenced IDO1 mRNA more efficiently than free siIDO1 or CS NPs (Figure 3F). Correspondingly, downregulation of IDO1 mRNA directly reduced IDO1 protein and downstream AhR protein expression, as evidenced by western blotting (Figure 3G,H). The AhR expression in Renca cells showed a consistent trend with that of IDO1, and both of them were markedly suppressed after CISE NPs treatment. These results indicated that CISE NPs can be endocytosed and escape from lysosomes into the cytoplasm, which effectively silences IDO1 mRNA expression and therefore downregulates IDO1 downstream protein expression and secretion.

Subsequently, we explored whether silencing IDO1 expression potentiated Sfn‐mediated cytotoxic effects. Apoptosis assays stained with Annexin V and PI confirmed that CISE NPs induced obvious early and late apoptosis (62.20 ± 1.56%) of Renca cells, which was more potent than CS NPs (44.57 ± 3.03%) or free Sfn (35.33 ± 1.79%) (Figure 3I,J). Modification of CD62E protein had a negligible effect on the cytotoxicity of CISE NPs. Identical results were obtained with calcein‐AM/PI live/dead staining, indicating that CISE NPs possessed greater tumor‐killing efficacy than other formulations (Figure S7, Supporting Information). Sfn, as a molecular targeted anti‐tumor agent, not only directly destroys tumor cells but also activates the body's adaptive immune response by inducing immunogenic cell death (ICD)‐triggered immunogenicity.^[^ ^41^ ^]^ Therefore, the ICD‐induction capacity of CISE NPs was analyzed in Renca cells by estimating damage‐associated molecular patterns including calreticulin (CRT), high‐mobility group box 1 (HMGB1), and adenosine 5′‐triphosphate (ATP). Treatment with Sfn‐containing nanoformulations (including CS, CIS, and CISE NPs) obviously elicited CRT exposure on the surface of tumor cells and reduced extracellular release of HMGB1 compared to treatment with PBS or Sfn alone (Figure 3K,L and Figure S8A,B, Supporting Information). In particular, CISE NPs‐treated Renca tumor cells showed the highest CRT exposure, HMGB1 release, and ATP secretion, indicating an intensified immunogenic death effect was induced (Figure S8, Supporting Information). These data indicate that CISE NPs can deliver functional siRNA to knock down IDO1 and induce cell apoptosis, thereby inducing ICD to trigger robust adaptive immunity.

### 2.3 PFD@Gel Optimized the TPME to Improve Perfusion and Relieve Hypoxia

**Figure 4:**
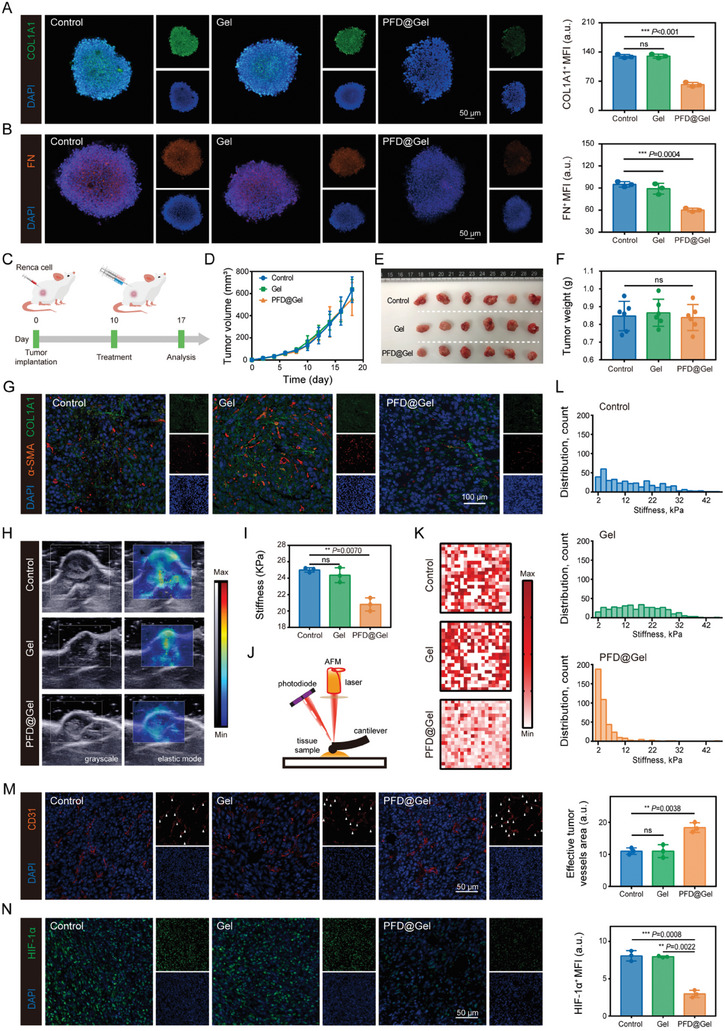
TPME remodeling effect induced by PFD@Gel. A) Representative CLSM images and relative quantification of COL1A1 (green) and B) FN (red) in Renca cells after varied treatments as indicated. C) Schematic of in vivo TPME assessment. D) Average tumor growth curves after varied treatment (n = 6). E) Representative digital photos and F) relative quantification of isolated tumor after varied treatments as indicated (n = 6). G) Immunofluorescence images showing COL1A1 (green) and α‐SMA (red) of tumor sections after varied treatments. H) Representative SWE images and I) relative quantification of tumor tissue after varied treatments (n = 3). J) Schematic of AFM measurement. K) Heat maps and L) histograms exhibited AFM force map results from varied groups. M) Immunofluorescence images and relative quantification of CD31 (red) expression in tumor sections (arrows indicate effective tumor vessels areas). N) Immunofluorescence images and relative quantification of HIF‐1α (green) in tumor sections. Data were expressed as means ± SD. Statistical differences were calculated using a two‐tailed unpaired Student's *t*‐test. ns, not significant, **P < 0.01, ***P < 0.001.

Increased secretion of collagen and other ECM components by tumor and stromal cells results in the surrounding tissue becoming stiffer and rigid, which not only limits drug penetration and immune cell infiltration but also provides the support needed for tumor cell growth and spread.^[^ ^6^, ^10^ ^]^ PFD is an oral anti‐fibrotic agent with anti‐fibrotic, anti‐inflammatory, and anti‐oxidant properties and has been widely used for the clinical treatment of idiopathic pulmonary fibrosis.^[^ ^31^, ^33^ ^]^ We further evaluated whether PFD could be used as an ECM modulator for physical microenvironment remodeling to potentiate targeted therapies for ccRCC. Renca multicellular spheroids (MCSs) were constructed to assess the ECM‐ameliorating effects of PFD in vitro. Immunofluorescence staining of Renca MCSs revealed that PFD@Gel suppressed the expression of collagen‐I (COL1A1) and fibronectin (FN), the most prominent components of the ECM (**Figure** **4**A,B). In contrast, the gel alone had negligible effects on COL1A1 or FN expression. The favorable cellular‐level modulatory efficacy of PFD@Gel motivated us to further evaluate its role in vivo. Mice bearing syngeneic RCC were treated with PBS, free gel, or PFD@Gel by intratumoral injection 10 days after tumor inoculation (Figure 4C). Average tumor growth curves revealed that neither the free gel nor PFD@Gel had a significant effect on tumor volume compared with the control group, as visualized in ex vivo tumor digital photos (Figure 4D–F; Figure S9, Supporting Information). The TPME conditions were assessed in ccRCC mice. Consistent with the in vitro analysis, immunofluorescence images showed that PFD@Gel treatment contributed to the potent suppression of ECM content, with a 37.6% downregulation of COL1A1 expression compared with the untreated control (Figure 4G; Figure S10, Supporting Information). The reduction in collagen fiber deposition in the PFD@Gel‐treated tumor tissues was confirmed by Masson's trichrome staining (Figure S11, Supporting Information). TGF‐β1 is a key mediator in the development of fibrosis and inflammation and plays critical roles in epithelial‐mesenchymal transition (EMT) and fibrosis formation.^[^ ^6^, ^10^ ^]^ As expected, PFD@Gel treatment downregulated TGF‐β1 protein expression in tumor tissues, which is consistent with previous reports that PFD induces collagen depletion mainly by suppressing TGF‐β1‐related signaling pathways (Figure S12, Supporting Information).

We conjecture that the remodeled ECM may, in turn, affect the mechanical properties of the TPME, of which tumor stiffness is an important element that cannot be ignored. To investigate the alteration in tumor mechanical features after different treatment formulations, tumor stiffness was monitored holistically and locally using shear wave elastography (SWE) and nanoscale atomic force microscopy (AFM),^[^ ^42^ ^]^ respectively. SWE is a highly reproducible technique that allows quantitative measurement of lesion stiffness based on estimates of shear wave velocity during ultrasound examination.^[^ ^43^ ^]^ The velocity information is available as a color map of tissue elasticity superimposed on a real‐time grayscale ultrasound image.^[^ ^44^ ^]^ Approximately 7 days after treatment, the group that received PFD@Gel treatment had a significantly reduced average elastic modulus compared with the other groups (Figure 4H,I). H&E staining and corresponding nanoscale AFM analysis were performed on snap‐frozen fresh tumor tissue sections after different treatments to determine tumor stiffness at the invasion front (Figure 4J; Figure S13, Supporting Information). Force maps showed that PFD@Gel treatment not only reduced tumor stiffness but also significantly attenuated the heterogeneity of stromal stiffness compared with the untreated and gel groups (Figure 4K,L). Benefiting from decreased tumor stiffness, the effective tumor vasculature area in the PFD@Gel‐treated tumors dramatically increased, which could be attributed to the reduced compression of vessels by the softened ECM (Figure 4M). However, the total area of the tumor vessels did not change significantly, indicating that PFD@Gel treatment did not affect tumor angiogenesis (Figure S14, Supporting Information). Consistently, assessment of tumor perfusion vessels using lectin‐labeled vascular endothelium (green) and CD31‐labeled total blood vessels (red) showed an obvious increase in the proportion of perfusion vessels within tumor treated with PFD@Gel, which could be attributed to the reduced compression of vessels by the softened ECM (Figure S15, Supporting Information). The increased effective tumor vasculature density is expected to improve oxygen supply within the tumor region, thus alleviating tumor hypoxia. Unsurprisingly, hypoxia fraction assessed by hypoxia‐inducible factor‐1α (HIF‐1α) expression of the viable ccRCC tissue was markedly reduced in PFD@Gel‐treated tumors (Figure 4N and Figure S16, Supporting Information). Meanwhile, applying oxyhemoglobin‐deoxyhemoglobin mode of photoacoustic imaging to detect tumor vascular saturated oxygen (sO_2_) levels revealed that tumors received PFD@Gel treatment displayed substantial increased sO_2_ signals, which further suggests alleviation of tumor hypoxia (Figure S17, Supporting Information). Altogether, the above results suggest that PFD@Gel treatment can remodel the TPME of ccRCC by reducing ECM deposition and blocking the TGF‐β1 signaling pathway, thus lowering tumor stiffness to increase vascular perfusion and alleviate hypoxia for favorable drug delivery and immune cell infiltration.

### 2.4 Effect of Biomimetic Hydrogel System on the Inhibition of ccRCC Relapse

**Figure 5:**
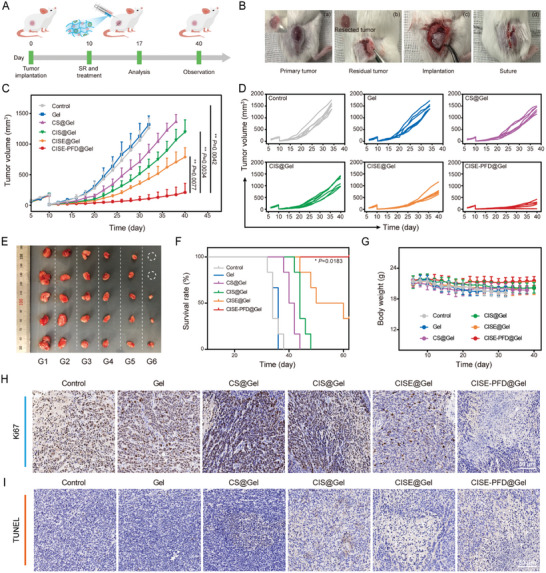
The CISE‐PFD@Gel system inhibits ccRCC recurrence after surgery. A) Schematic of animal experiment design. B) Digital photos displaying recurrent ccRCC model construction and treatment. C) Average and D) individual tumor growth curves in varied groups (n = 6). E) Representative digital photos of isolated tumors on 17 day post treatment. F) Survival and G) body weight fluctuation curves of tumor‐bearing mice in varied groups (n = 6). H) Immunohistochemical images of Ki67 and I) TUNEL in tumor sections from each group. G1, Control; G2, Gel; G3, CS@Gel; G4, CIS@Gel; G5, CISE@Gel; G6, CISE‐PFD@Gel. Data were expressed as means ± SD. Statistical differences were calculated using two‐tailed unpaired Student's *t*‐test and Log‐rank test. *P < 0.05, **P < 0.01.

To examine whether PFD@Gel‐elicited TPME remodeling could potentiate CISE NP‐triggered anti‐tumor efficacy in vivo, we constructed a postoperative ccRCC recurrence model. On the 10th day after inoculation with Renca cells, a part of the visible tumor was surgically removed and treated with different gel formulation combinations, including the control, free gel, CS@Gel, CIS@Gel, CISE@Gel, and CISE‐PFD@Gel (**Figure** **5**A,B). Equivalent drug doses were kept consistent across the groups (siIDO1 dosage, 1 mg kg^−1^; Sfn dosage, 10 mg kg^−1^; and PFD dosage, 16 mg kg^−1^). Residual tumor growth and body weight were observed and recorded until the mice reached a tumor volume of 1500 mm^3^ or exhibited cachexia. Tumor relapse appeared rapidly in the untreated and free gel‐treated groups, with a median survival time of 33.8 and 35.4 days after surgery, respectively (Figure 5C–F). Mice receiving CISE@Gel exhibited a prominent decline in tumor growth, which was far more effective than CS@Gel and CIS@Gel, which may be attributed to the superimposed effects of IDO1 silencing and CD62E‐mediated tumor targeting. Notably, treatment with CISE‐PFD@Gel exhibited a superior capacity to decelerate postoperative ccRCC recurrence, with a tumor inhibition rate of 83.3% among all the groups, and all mice survived for more than 60 days. Compared to the slight decrease in mouse body weight due to tumor burden in the control and free gel groups, mice in the other treatment groups showed almost no significant difference in body weight during the observation period (Figure 5G). On day 17, the tumors from all groups were isolated and subjected to immunohistochemical analysis. Consistent with the trend in tumor growth kinetics, CISE‐PFD@Gel treatment caused severe damage to tumor tissues and greatly inhibited tumor cell proliferation, as revealed by the Ki67 and TUNEL indices (Figure 5H,I). Additionally, we explored whether such a combined mechano‐immunometabolic therapy strategy could potentially be used for other renal cancer types by employing murine RAG syngeneic renal cell carcinoma as the model. Similar to previous observations, CISE‐PFD@Gel also exhibited remarkable therapeutic efficacy against murine postsurgical residual RAG renal cell carcinoma and significantly reduced the tumor growth rate compared to controls (Figure S18, Supporting Information). Moreover, we also investigated whether the combination therapy would be effective against other strains of tumor‐bearing mice. In a postoperative B16‐F10 melanoma model, CISE‐PFD@Gel treatment inhibited residual tumor growth to a great extent (Figure S19, Supporting Information). These results successfully proved that our mechano‐immunometabolic therapy strategy could be applied not only to kidney cancer but also extended to other types of solid tumors.

Considering that biosafety is a prerequisite for effective treatment, we further evaluated the effect of CISE‐PFD@Gel treatment on major organs, including the liver and kidney function indicators in mice. Serological (blood urea nitrogen, albumin, alanine aminotransferase, and aspartate transaminase) and hematological (leukocyte, red blood cell, mean corpuscular volume, hemoglobin, mean corpuscular hemoglobin concentration, and platelet) indices were all within the normal range; H&E staining of the major organs (lung, liver, spleen, kidney, and heart) did not show any pathological structural abnormalities, indicating that ccRCC mice could tolerate CISE‐PFD@Gel treatment (Figure S20, Supporting Information).

### 2.5 Metabolic Blockade Synergized the TPME Remodeling to Boost ccRCC Immune Activation

**Figure 6:**
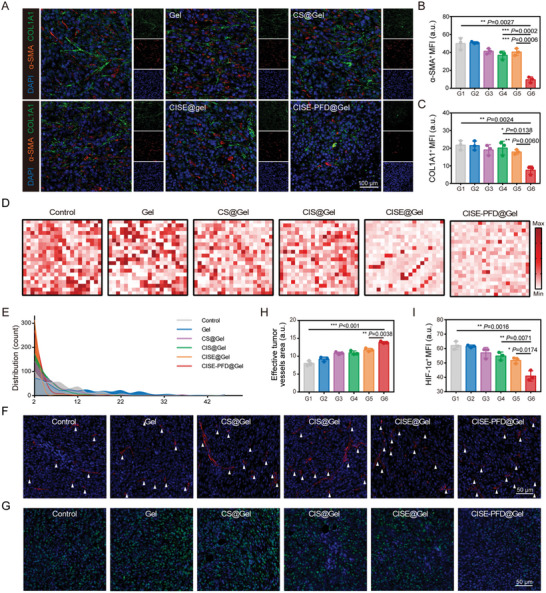
CISE‐PFD@Gel system remodels TPME in recurrent ccRCC. A) Immunofluorescence images and relative quantification of B) COL1A1 (green) and C) α‐SMA (red) of tumor sections after varied treatment. D) Heat maps and E) histograms exhibited AFM force map results after varied treatments. F) Immunofluorescence images and H) relative quantification of CD31 (red) expression in tumor sections (arrows indicate effective tumor vessels areas). G) Immunofluorescence images and I) relative quantification of HIF‐1α (green) in tumor sections. G1, Control; G2, Gel; G3, CS@Gel; G4, CIS@Gel; G5, CISE@Gel; G6, CISE‐PFD@Gel. Data were expressed as means ± SD. Statistical differences were calculated using a two‐tailed unpaired Student's *t*‐test. *P < 0.05, **P < 0.01, ***P < 0.001.

To further elucidate the potential mechanisms underlying the potent anti‐tumor efficacy of the CISE‐PFD@Gel therapeutic regimen, tumor tissues isolated from mice treated with various formulations were collected to detect ECM variations. COL1A1, the predominant collagen deposited during chronic fibrosis, is a major contributor to the mechanical properties of the ECM.^[^ ^6^, ^13^ ^]^ Immunofluorescence staining revealed a significant reduction in collagen fiber deposition and activated CAFs in gel formulations loaded with PFD, which was consistent with the results of Masson's trichrome staining (**Figure** **6**A–C; Figure S21, Supporting Information). The stiffness of the peripheral invasive regions of the tumor was mechanically tested using AFM, and force maps of these regions were constructed in parallel. We found reduced solid stress of tumor tissues in the recurrent ccRCC tumors treated with CISE‐PFD@Gel, which obviously shifted to a lower stiffness compared with the control group (Figure 6D). Frequency plots of the corresponding ECM regions showed that the stiffness distribution in the CISE‐PFD@Gel‐treated group was smaller and narrower than that in the other groups (Figure 6E). Importantly, benefiting from the decreased tumor stiffness, the effective tumor vasculature area of the CISE‐PFD@Gel‐treated group was dramatically increased, which is consistent with our previous experiments (Figure 6F,H). The increased perfusion vasculature further ameliorated hypoxia in the tumor region, as evidenced by immunofluorescence of the classical hypoxia marker, HIF‐1α (Figure 6G,I). These results indicated that the biomimetic hydrogel system lowered ECM stiffness in recurrent ccRCC by consuming collagen deposition, which fosters vascular opening and perfusion while ameliorating tumor hypoxia.

**Figure 7:**
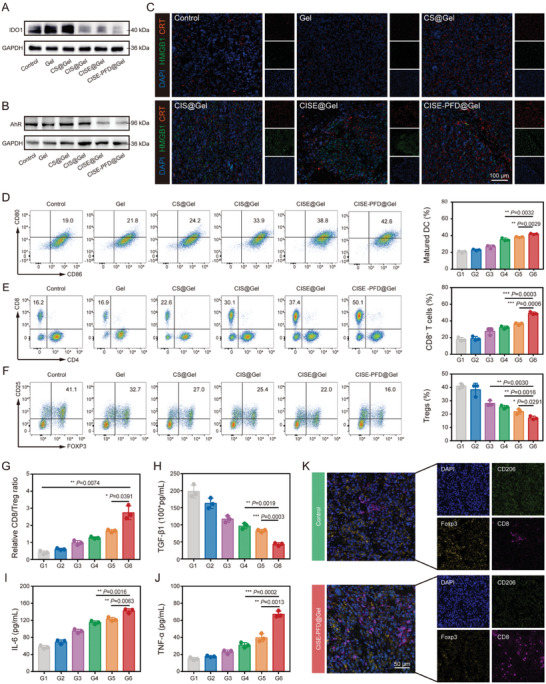
Metabolic blockade triggers robust systemic immune responses. A) Western blotting of IDO1 expression within tumors after varied treatment. B) Western blotting of AhR expression within tumors after varied treatment. C) Immunofluorescence images of tumor slices showing HMGB1 (green) and CRT (red) expression after varied treatment. D–F) Typical flow cytometric images and relative quantification of infiltrated D) mDCs (CD80^+^CD86^+^), E) CD8^+^ T cells and F) Tregs (CD25^+^Foxp3^+^) within tumor tissues. G) The ratio of CD8^+^ T cells versus Tregs. H–J) Serum cytokine levels of H) TGF‐β1, I) IL‐6 and J) TNF‐α from each group. K) Representative polychromatic immunofluorescence images of tumor sections showing DAPI (blue), CD206 (green), Foxp3 (orange), and CD8 (red) cells infiltration in control and CISE‐PFD@Gel treated groups. G1, Control; G2, Gel; G3, CS@Gel; G4, CIS@Gel; G5, CISE@Gel; G6, CISE‐PFD@Gel. Data were expressed as means ± SD (n = 3). Statistical differences were calculated using a two‐tailed unpaired Student's *t*‐test. *P < 0.05, **P < 0.01, ***P < 0.001.

Since vascular function and tissue oxygenation correlate with increased immune cell infiltration and activity, we investigated whether the potent anti‐tumor response produced by CISE NPs in normalized TPME depends on tumor immunogenicity. The function of siIDO1 was first estimated using immunofluorescence and western blotting analyses. Consistent with the in vitro cellular results, CISE@Gel treatment significantly suppressed IDO1 protein expression in ccRCC tissues (**Figure** **7**A; Figure S22, Supporting Information). Generally, IDO1 signaling foster a tumor immunological microenvironment that is defective in recognizing and eradicating cancer cells by depleting Trp to generate Kyn, which in turn upregulates the immune‐tolerant cells levels within tumor tissues via activating AhR pathway. As expected, siIDO1‐containing gel groups downregulated the Kyn/Trp ratio, especially in the CISE‐PFD@Gel‐treated group, implying that the Trp metabolic pathway was successfully disrupted (Figure S23A, Supporting Information). Meanwhile, CISE‐PFD@Gel‐treated Renca renal carcinoma tissues also exhibited significantly lower AhR expression and showed better inhibition efficacy than that of the CISE@Gel group, which may be attributed to PFD‐elicited TPME normalization promoted more CISE NPs to be phagocytosed by tumor cells (Figure 7A,B; Figure S23B, Supporting Information). Additionally, because blocking the IDO1 pathway enhanced the cytotoxic effects of Sfn in vitro, we speculated whether it could also enhance tumor immunogenicity to induce an adaptive immune response. Significantly abundant CRT and HMGB1 fluorescence signals were detected in tumor sections after CISE‐PFD@Gel treatment, indicating that intense ICD was elicited (Figure 7C; Figure S24, Supporting Information). This fortified ICD effect transforms into a powerful anti‐tumor immunity by promoting DC maturation and sequentially eliciting immune responses.

Next, we investigated how CISE‐PFD@Gel intervention affected the tumor immune microenvironment in postoperative ccRCC using FCM. The mature dendritic cell (mDCs) ratio in the CIS@Gel, CISE@Gel, and CISE‐PFD@Gel groups was obviously increased compared with that in the control and free gel groups, confirming that IDO1 blocking indeed elevated the DAMP signals elicited by Sfn (Figure 7D). Notably, the percentage of immune‐positive cells including CD8^+^ T cells and M1‐TAMs (CD86^hi^CD11b^+^F4/80^+^) in CISE‐PFD@Gel‐treated mice displayed the highest infiltration as compared with that in other groups (Figure 7E; Figure S25, Supporting Information). Conversely, immune‐negative cells, such as Tregs (CD4^+^CD25^+^Foxp3^+^) and M2‐TAMs (CD206^hi^CD11b^+^F4/80^+^), were significantly reduced in the groups containing Sfn and siIDO1, especially in the CISE‐PFD@Gel group, suggesting that PFD‐mediated TPME remodeling may contribute to attenuating the postoperative immune suppression status in ccRCC (Figure 7F; Figure S25, Supporting Information). Indicators of immune activation, such as the ratios of CD8^+^ T/Treg and M1‐TAMs/ M2‐TAMs were all highly elevated in the CISE‐PFD@Gel group (Figure 7G; Figure S25, Supporting Information). This shift to a positive immune equilibrium was further confirmed by polychromatic immunofluorescence staining analysis, as displayed by the spatial distribution of increased CD8 expression and downregulated CD206 and Foxp3 expression within tumor tissues after CISE‐PFD@Gel treatment (Figure 7K). The CISE‐PFD@Gel‐treated mice secreted the highest levels of tumor necrosis factor‐α (TNF‐α) and IL‐6 in the serum, but the opposite trend was observed for TGF‐β1, which again verified the efficacious immune responses triggered by our biomimetic hydrogel system (Figure 7H–J). Our combination strategy of TPME remodeling synergized with immune‐metabolic blockade can establish an inflammatory tumor immune niche via disrupting the IDO1/Kyn/AhR axis‐mediated immune escape, which exerts effective tumoricidal immune activity to inhibit postsurgical ccRCC recurrence.

### 2.6 Biomimetic Hydrogel System Hinderd EMT to Combate Lung Metastases

**Figure 8:**
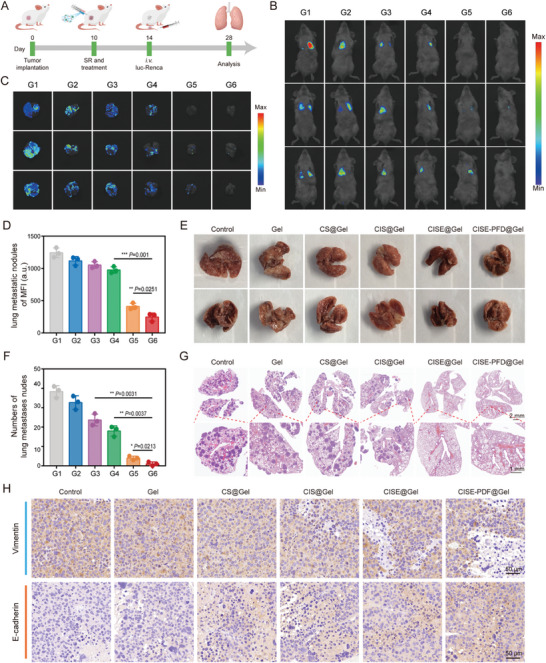
CISE‐PFD@Gel system combats lung metastases via EMT suppression. A) Schematic of lung metastases model treatment. B) Representative bioluminescence images of tumor‐bearing mice after varied treatments. C) Bioluminescence images and D) relative quantification of isolated lung tissues from each group. E) Representative digital photos of the ex vivo lung tissues. F) Quantification of pulmonary metastasis nodules in different groups. G) Representative H&E staining of lung tissues from each group. H) Immunohistochemical images of vimentin and E‐cadherin in tumors sections after varied treatments as indicated. G1, Control; G2, Gel; G3, CS@Gel; G4, CIS@Gel; G5, CISE@Gel; G6, CISE‐PFD@Gel. Data were expressed as means ± SD (n = 3). Statistical differences were calculated using a two‐tailed unpaired Student's *t*‐test. *P < 0.05, **P < 0.01, ***P < 0.001.

Approximately 30% of ccRCC patients experience recurrence or metastasis after radical surgery, and the 5‐year survival rate of patients with metastatic RCC is less than 20%. Considering the crucial role of metastasis in the prognosis of ccRCC patients after surgery, we further investigated the antimetastatic ability of the CISE‐PFD@Gel therapeutic strategy. A mouse model postoperative ccRCC recurrence was established and treated according to previously described subgroups. On day 14 after primary tumor inoculation, luciferase‐transfected Renca (luc‐Renca, 1 × 10^5^) cells were intravenously administered in mice to construct a distant lung metastasis model of ccRCC (**Figure** **8**A). Lung metastasis of luc‐Renca tumors was tracked using in vivo bioluminescence imaging and lung tissues were harvested for immunohistochemical staining at the end of the observation period. In mice treated with PBS, free gel, or CS@Gel, marked bioluminescence signals and metastatic nodules were observed in the lung region 18 days after treatment, which was confirmed by H&E staining of isolated lung sections (Figure 8B–G). In contrast, CISE‐PFD@Gel treatment inhibited lung metastasis more effectively than CIS@Gel or CISE@Gel and resulted in the lowest lung metastatic tumor formations, as evidenced by quantifying luciferase intensity and counting nodule numbers (Figure 8D,F). EMT, an inflammatory process, is generally regarded as the initiator of tumor cell invasion.^[^ ^45^ ^]^ In particular, vimentin (a mesenchymal‐like cell‐cell adhesion molecule) and E‐cadherin (an epithelial‐like cell‐cell adhesion molecule) are typical EMT markers.^[^ ^46^ ^]^ High vimentin expression indicates increased tumor cell aggressiveness, whereas decreased E‐cadherin expression suggests high tumor metastatic potential. As expected, lung tissues showed lower vimentin expression and more E‐cadherin was present in the CISE‐PFD@Gel‐treated groups than in the other groups (Figure 8H). These results demonstrate that our tailor‐prepared biomimetic hydrogel system, which combines TPME remodeling and immune metabolic blockade, can be used to prevent EMT in combating ccRCC metastasis after surgery.

## 3 Conclusion

ccRCC is one of the most common malignant tumors of the urinary system, with an annual increase of ≈2% worldwide over the past two decades.^[^ ^1^, ^47^ ^]^ Surgical resection remains the first‐line clinical intervention for patients with localized ccRCC.^[^ ^4^, ^30^ ^]^ However, ≈20% of ccRCC patients experience postoperative relapse. Recurrent ccRCC presents a major clinical challenge, and patients in whom localized therapy fails tend to have poor prognosis.^[^ ^48^ ^]^ Although combination regimens of targeted therapy and immunotherapy have improved the survival of ccRCC patients, the prognosis of patients with advanced ccRCC, including recurrent ccRCC, remains poor.^[^ ^49^, ^50^ ^]^ A thorough exploration of treatment‐resistant ccRCC could deepen our understanding of tumorigenesis and development mechanisms, and more importantly, contribute to the development of more effective ccRCC treatment regimens.

Here, based on bioinformatics analysis derived from TCGA database and clinical sample validation, we identified that altered tumor mechanics in the TPME and abnormal cellular IDO1 metabolism are two major factors that contribute to ccRCC aggression and compromise treatment efficacy. Therefore, we developed a topical injectable biomimetic hydrogel‐mediated mechanical immunometabolic therapeutic strategy to inhibit ccRCC recurrence after surgical resection. Specifically, PFD, an FDA‐approved clinically wonder drug for the treatment of idiopathic pulmonary fibrosis, was first introduced to inhibit TGF‐β1 and its downstream signals, thereby reducing collagen deposition and remodeling stiff ECM to become soft. To track and eliminate tumor microsatellites and infiltrating ccRCC cells within the resection cavity, a nanomodulator called CISE NPs, carrying Sfn and siIDO1, was synthesized. Benefiting from CD62E protein‐mediated leukocyte adhesion, the nanomodulator could travel by leukocyte‐hitchhiking to achieve higher accumulation in postoperative inflamed residual tumors. Subsequently, Sfn in the nanomodulator elevated the expression of damage‐associated molecular patterns in dead tumor cell‐related antigens, which promoted the activation of infiltrating CTLs. Meanwhile, siIDO1 silencing of the IDO1 metabolic pathway alleviated immune brakes associated with immunonegative cells, such as Tregs and M2‐TAMs, which further reinforces anti‐tumor immunity. More importantly, intracavity drug delivery via the biomimetic hydrogel provided a safe, convenient, and efficient approach that can be seamlessly incorporated into clinical surgical debulking without requiring additional surgery to cascade the stimulation of hot tumoricidal immunity.

We successfully developed a unique biomimetic hydrogel system, CISE‐PFD@Gel, capable of mechanically remodeling the ECM, blocking immune metabolism, and intensifying tumor immunogenicity to generate inflamed immune niches for optimizing anti‐tumor immune responses. Specifically, this system exhibited remarkable targeting toward the residual tumor cells around the resected cavity, which not only effectively suppressed ccRCC postoperative recurrence but also wholly regressed lung metastasis with negligible systemic toxicity. Our meticulously designed mechano‐immunometabolic therapy provides an alternative for postoperative adjuvant therapy in ccRCC patients undergoing surgical resection. The potential clinical translation of this strategy, coupled with its convenience and efficacy, may enrich the arsenal of TME remodeling strategies for better cancer immunotherapeutic efficacy.

## 4 Experimental Section

### Human Tumor Specimens

This study was approved by the Ethics Committee of Shanghai Tenth People's Hospital. All samples were obtained with written informed consent and collected using a standard protocol approved by the review committee of the Shanghai Tenth People's Hospital (Approve no. SHSY‐IEC‐4.1/19‐211/01).

### Analysis of TCGA Data

IDO1 and TGF‐β1 expression in human renal cell carcinoma versus counterpart normal tissues were analyzed from TCGA databases and the genotype‐tissue expression were determined by gene expression profiling interactive analysis (http://gepia.cancer‐pku.cn/detail.php?gene = &clicktag = boxplot). Survival analysis of RCC patients with high and low IDO1 or TGF‐β1 protein expression was derived from GEPIA (http://gepia.cancer‐pku.cn/detail.php?gene = &clicktag = survival).

### Materials

The siIDO1 (sense strand 5′‐GCAAUAGUAGAUACUUACA‐3′), the negative control siRNA (a scrambled sequence consisting of the sense strand 5′‐UUCUCCGAACGUGUCACGUTT‐3′) and the Cy3‐siRNA were synthesized by Genomeditech Biotechnology Co., Ltd. CaCl_2_ and Na_3_PO_4_·12H_2_O were obtained from Sinopharm Chemical Reagent Co., Ltd. DOPA was obtained from Shycbio Biotechnology Co., Ltd. Sfn was obtained from Targetmol (Shanghai, China). The CD62E protein was purchased from ABclonal Biotechnology Co., Ltd. The bovine fibrinogen, thrombin, and LysoTracker Green lysosomal fluorescent probe were purchased from Yeasen Biotechnology Co., Ltd. PFD was purchased from Yuanye Biotechnology Co., Ltd. Calcein‐AM/PI Double Staining Kit was purchased from Dojindo. The DAPI staining solution, cell counting kit‐8, ATP assay kit, and D‐luciferin potassium salt were purchased from Beyotime (Shanghai, China). The mouse IL‐6, TGFβ1, and TNF‐α ELISA kits were obtained from Liankebio Biotechnology Co., Ltd.

### Cell Lines and Animals

Renca and RAG cells were originally obtained from the American Type Culture Collection (ATCC). Luciferase‐labeled Renca cells (luc‐Renca) were obtained from OBiO Technology Corp., Ltd (Shanghai, China). Renca and luc‐Renca cells were maintained in RPMI‐1640 (Gibco), RAG cells were maintained in MEM (Gibco), supplemented with fetal bovine serum (10%, HyClone), penicillin‐streptomycin (1%, HyClone), and non‐essential amino acids (1%, Gibco) incubated at 37 °C and 5% CO_2_. Balb/c mice (male, 6–8 weeks) were ordered from GemPharmatech Co., Ltd (Shanghai, China). Mice were housed in an SPF‐grade pathogen‐free facility with a light/dark cycle of 12 h at 20 ± 3 °C and relative humidity of 40% to 70%. All animal experiments were performed according to protocols following the policies of the State Ministry of Health and were approved by the Experimental Animal Center of the Shanghai Tenth People's Hospital (SHDSYY‐2023‐3123).

### Western Blotting Procedures

The obtained protein was quantified by a bicinchoninic acid protein assay kit (Epizyme, China). The equivalent protein was mixed with the protein loading buffer (Epizyme, China) and boiled (100 °C, 10 min), followed by electrophoresis on SDS‐PAGE gels and the transfer of proteins onto a nitrocellulose membrane (Boster, China). After blocking with nonfat milk (5%) for 2 h, the membranes were incubated with diluted anti‐IDO1 antibody (CST, dilution 1:1000), anti‐TGF‐β1 antibody (CST, dilution 1:1000), anti‐HIF‐1α antibody (Bioss, dilution 1:1000), and anti‐GAPDH antibody (Genomeditech, dilution 1:1000) at 4 °C overnight followed by incubation with anti‐rabbit lgG‐HRP secondary antibody (CST, dilution 1:5000) for 1 h at room temperature. Electrochemiluminescence imaging was captured by an automatic chemiluminescence image analysis system (USA, Amersham Imager 600).

### RT‐qPCR Assay

The IDO1 gene‐silencing efficiency of siRNA was investigated by RT‐qPCR. Briefly, Renca cells were transfected with different siRNA by Lipofectamine 3000, and incubated for 24 h at 37 °C. Total RNA was isolated in an RNA lysis buffer and cDNA was synthesized with the PrimeScript II 1st strand cDNA synthesis kit (Takara, Catalog No.6210A) following the manufacturer's protocol. The provided siIDO1 Primers: F‐5′‐GGGCUUCUUCCUCGUCUCU‐3′, R‐5′‐AGAGACGAGGAAGAAGCCC‐3′; F‐5′‐GUUCUAGAAGGAUCCUUGA‐3′, R‐5′‐UCAAGGAUCCUUCUAGAAC‐3′; F‐5′‐GCAAUAGUAGAUACUUACA‐3′, R‐5′‐UGUAAGUAUCUACUAUUGC‐3′. GAPDH Primers: F‐5′‐ACAGTCAGCCCGCATCTTCTT‐3′, R‐5′‐CGACCAAATCCGTTGACTC‐3′. The mRNA expression level was analyzed and compared with that for GAPDH on a QuantStudio Dx detection system quantified via the 2^−△△CT^ method.

### Preparation and Characterizations of CISE NPs

The anionic lipid coating CaP cores were prepared by a water‐in‐oil micro‐emulsion method. Briefly, CaCl_2_ (300 µL, 500 mM) with siIDO1 or Cy3‐siRNA (100 µL, 2 mg mL^−1^) was dispersed in Cyclohexane/Igepal CO‐520 solution (15 mL) to form a well‐dispersed water‐in‐oil reverse micro‐emulsion. The phosphate part was prepared by Na_2_HPO_4_ (300 µL, 25 mM) in a separate oil phase (15 mL), and DOPA (200 µL, 20 mg mL^−1^) in chloroform was added to the phosphate phase. After mixing the above two solutions for 20 min, absolute ethanol (30 mL) was added to the micro‐emulsion, and the mixture was centrifuged (12000 g, 20 min) to remove cyclohexane and surfactant to obtain the CaP‐siIDO1 (CI) NPs. After being extensively washed with ethanol three times, the pellets were dissolved in chloroform (1 mL) and stored in a glass vial for further modification. Then, CI NPs (500 µL) was mixed with DMPC, cholesterol, DSPE‐PEG‐2000 and Sfn (mass ratio of 3:1:1:0.5) to obtain Cap‐siIDO1‐Sfn (CIS) NPs under evaporation. To achieve active tumor‐targeting, the CIS NPs solution was co‐incubated with His‐tagged CD62E protein at 37 °C for 1 h, followed by 4 °C overnight to yield CD62E modified CIS (CISE) NPs. UV‐vis spectrum was recorded by UV–vis spectrophotometer (PE Lambda 950). The zeta potential and hydrated particle size were determined by a laser granularity sizer (Zetasizer Nano ZS90). Transmission electron microscopy (TEM, FEI Tecnai G2 F30) was used to observe the morphology and elemental composition of CISE NPs. Prepared samples (10 µL) were loaded into agarose gel (2 wt%) with GelRed, and an electrophoresis assay detected the loading of siIDO1 in CISE NPs. Electrophoresis was run in 1×Tris‐Acetate‐EDTA buffer (80 V, 25 min) and visualized by an ultraviolet transilluminator and a digital imaging system (IS‐2200, Alpha Innotech). The CD62E protein anchored on CISE NPs was detected by SDS‐PAGE protein analysis. SDS‐PAGE Gel added with samples was run in 1×Tris‐Glycine electrophoretic buffer (120 V, 50 min) followed by staining with Coomassie brilliant blue (Yeasen), then washed in acetic acid solution (10%, 2 h) to visualize by a light transilluminator and a digital imaging system.

### Preparation and Characterization of the Biomimetic Hydrogel System

The CISE‐PFD@Gel was formed from the interaction of bovine fibrinogen and thrombin. Briefly, fibrinogen (50 mg) was dissolved in PBS (1 mL) under ultrasound (3 min), then placed in water bath (37 °C) and gently stirred (30 min). Predetermined CISE NPs and PFD (0.6 mg mL^−1^) were then added into fibrinogen solution to form precursor solution 1. The thrombin (500 IU mL^−1^) was dissolved in PBS containing NaCl (26.3 mg L^−1^) and CaCl_2_ (7.7 mg L^−1^) to form precursor solution 2. Precursor solution 1 and 2 were mixed (volume ratio 2:1) to form CISE‐PFD@Gel. The synthesized biomimetic hydrogel was then tested for injectability using a syringe with a 25‐gauge needle. The morphology of hydrogel was detected by Cryo‐SEM (SU8010). Rheological measurements were performed on a rheometer (TA Instruments AR 2000). For in vitro degradation behavior, hydrogel was incubated with PBS kept at 37 °C and the residual gels were weighed and recorded. For in vivo biodegradable and biocompatible behavior, the hydrogel was injected into the abdomen of Balb/c mice (200 µL sample per mice) to observe gel degradation, and mice skin tissues were taken on days 0, 3, 7, 14, 21, and 28 for H&E staining to assess the tissue biocompatibility. Meanwhile, mice were euthanized at pre‐set time points, and the hydrogel in each mouse was gently homogenized and aliquoted to determine the remaining PFD, Sfn and siRNA in the gel. The Sfn and PFD release kinetics were assessed by UV–vis spectroscope (PE Lambda 950), while the Cy3‐siRNA was determined by microplate reader (Multiskan FC).

### Cellular Uptake and Lysosomal Escape

Renca cells were seeded in 6‐well plates. After being cultured overnight, the cells were treated with Cy3‐labelled CISE NPs (20 µg mL^−1^) for varied times. The endocytosis of CISE NPs was qualitatively and quantitatively detected by CLSM and FACS (BD, FACS Canto, the USA). To evaluate endo/lysosomes escape effect, Renca cells were seeded in confocal dishes and cultured until cell attachment. After incubating with Cy3‐labelled CISE NPs for varied times, the cells were stained with LysoTracker Green (50 nM, 1 h) and observed under CLSM (Germany, CarlZeiss LSM900).

### Cell Viability Assessment

The in vitro anti‐tumor efficacy of CISE NPs was evaluated by CLSM and FCM. Briefly, Renca cells were cultured in 24‐well plates. The cells were co‐incubated with varied nanoformulation for 12 h. For CLSM observation, the adherent cells were stained with Calcein‐AM/PI double staining kit (Dojindo, Japan) according to instructions. For FCM apoptosis analysis, the cells were collected and stained with Annexin V‐FITC and PI dye.

### ICD Evaluation

To determine the CISE NPs‐induced ICD, the surface expression of CRT and extracellular release of HMGB1 were examined in Renca cells. Typically, Renca cells (1 × 10^5^ cells per well) were seeded in confocal dishes (Solarbio). Then, the cells were treated with PBS, Sfn, CS, CIS and CISE NPs, respectively. After incubation for 12 h, the cells were collected and incubated with anti‐HMGB1 (Abcam, dilution 1:200) and anti‐CRT (Abcam, dilution 1:100) for 1 h followed by stained with goat anti‐rabbit IgG H&L (Abcam, dilution 1:200) for 1 h. Before observed under CLSM, cells were stained with DAPI for 10 min. The extracellular secretion of ATP was quantified using an ATP assay kit (Beyotime).

### Multicellular Spheroids Assay

Renca cells (1000 cells) were plated on 96‐well ultra‐low attachment plates (Corning) in complete media. Media was refreshed every other day by carefully aspirating 50% of the well volume and replacing it with fresh complete media. When tumor spheroids diameter grows ≈400 µm, PBS, Gel, PFD@Gel (PFD dosage: 100 µg mL^−1^) were added into plate. After co‐cultured for 24 h, tumor spheroids were successively immobilized, blocked, incubated overnight at 4 °C with primary antibody and conjugated with secondary antibody for 1 h room temperature. Cell nuclei were stained with DAPI. The antibodies involved including anti‐collagen I antibody (Abcam, dilution 1:1500), anti‐fibronectin antibody (Abcam, dilution 1:50) and Alexa Fluor 488‐conjugated affinipure goat anti‐rabbit IgG (H + L) (Jackson, dilution 1:500).

### Mechanical Remodeling of TPME

To assess the TPME remodeling effect of PFD@Gel, Renca cells (1 × 10^6^ cells) were subcutaneously injected into the right flank of the Balb/c mice (6‐8 weeks) to establish ccRCC tumor model. When tumor volume reached 200–300 mm3, mice were randomized and divided into three groups: (1) Control, (2) Gel, and (3) PFD@Gel (n = 6). The tumor size and body weight of mice were measured every other day after treatment. The tumor volume (mm^3^) calculation formula: V = 1/2 × a × b^2^. When tumor volume exceeded 1500 mm^3^ or cachexia signs appeared, mice were euthanized. To evaluate ECM protein expression, tumor slices were collected on day 7 after treatment and subjected to Masson's trichrome staining and immunofluorescence staining. The antibodies involved including anti‐α‐SMA (CST, dilution 1:50), anti‐COL1A1 (CST, dilution 1:100), anti‐HIF‐1α (Abcam, dilution 1:50) and anti‐CD31 (Abcam, dilution 1:100). For tumor stiffness measurement, an Aixplorer ultrasonic scanner (SuperSonic Imagine) with linear transducer (4‐15 MHz) was used to acquire the B‐mode and SWE‐mode images of tumors. The test was performed by a senior radiologist according to a standardized and reproducible protocol. The mean elasticity (E_mean_) and standard deviation (SD), automatically calculated and visualized by the system, were selected for analysis. Meanwhile, fresh tumor sections (30 µm) were collected for AFM measurement. Considering the heterogeneity of the tumors, tissue stiffness was always measured in the tumor‐invasive regions to reduce intra‐tumoral variability. The spring constant of cantilever set as 0.03 N m^−1^ and cantilevers were tapped on the tumor stroma. The Hertz model was used to determine the elastic properties of the tissue. To evaluate tumor vascular perfusion, FITC‐labeled tomato lectin (150 µL, 1 mg mL^−1^; Vector Laboratories) was injected into tumor‐bearing mice via the tail vein. Mice were anesthetized 15 min later and perfused by intracardiac injection with PBS (1% paraformaldehyde) to remove circulating and lectin. Tumor tissues were stained with anti‐mouse CD31 antibody (BD Biosciences) and secondary Alexa Fluor 647‐conjugated goat anti‐rat IgG, then visualized under an Olympus FV‐1000 confocal microscope. Photoacoustic imaging (VEVO LAZR‐X) was utilized to assess the hypoxia‐relief capacity of PFD@Gel in vivo. Blood oxygen saturation was the ratio of photoacoustic signal intensity of oxyhemoglobin (λ = 850 nm) and deoxyhemoglobin (λ = 750 nm). Then, the photoacoustic signals of regions of interest were measured and analyzed. Tumor tissues were collected at the end of photoacoustic signal acquisition and analyzed with a western blot assay to detect HIF‐1α protein expression.

### Tumor Models and Treatment Protocols

To evaluate the anti‐tumor effect of CISE‐PFD@Gel, Renca cells (1 × 10^6^ cells) or RAG cells (2.5 × 10^6^ cells) were subcutaneously injected into the right flank of the Balb/c mice (6‐8 weeks) to establish ccRCC tumor model. When tumor volume reached about 200–300 mm^3^, tumor tissues were removed with sterile instruments, leaving about 40–60 mm^3^ to mimic residual tumors. Then mice were randomized divided into six groups (6 mice per group) including (1) Control, (2) Gel, (3) CI@Gel, (4) CIS@Gel, (5) CISE@Gel and (6) PFD@Gel. PBS or different formulated hydrogel (150 µL, PFD dosage: 20 mg kg^−1^, Sfn dosage: 10 mg kg^−1^ and siIDO1 dosage: 0.85 mg kg^−1^) were injected into the tumor resection cavity after surgery. The recurrent tumor size and body weight of mice were measured every other day during observation. To assess the anti‐lung metastasis effect of CISE‐PFD@Gel, the recurrent ccRCC tumor model was established and treated as previously described. On day 14 after tumor implantation, luc‐Renca cells (1 × 10^5^ cells) were intravenously injected into the tumor‐bearing mice. The metastatic nodules were assessed and lung tissues were isolated on day 18 post treatment for immunohistochemistry staining.

### Cytokine Analysis and Measurements of Kyn and Trp

The serum levels of TGF‐β1, IL‐6 and TNF‐α in tumor tissues were determined using ELISA assay kits (Liankebio) following the manufacturer's instructions. To evaluate changes in Trp and Kyn contents after treatment, tumor tissues were collected on day 7 post treatment and homogenized in saline at 4 °C. Then, 10% trichloroacetic acid was added, and the supernatant was measured for Kyn (360 nm) and Trp (280 nm) under HPLC with a mobile phase of ammonium acetate with methanol (10%, w/v), respectively.

### FCM Analysis

The tumor tissues were digested into single‐cell suspension, blocked with anti‐CD16/CD32 (eBioscience) at 4 °C for 10 min and stained with varied diluted fluorochrome‐conjugated antibodies. The antibodies involved including CD45‐BV510 (Biosciences, Catalog No.563204), CD3‐PE‐Cy7 (Biosciences, Catalog No.552774), CD4‐FITC (Biosciences, Catalog No.553729), CD8‐PerCP‐Cy5.5 (Biosciences, Catalog No.567597), Foxp3‐PE (Biosciences, Catalog No.566881), CD11b‐APC (Biosciences, Catalog No.553312), CD11c‐APC (Biosciences, Catalog No.550261), MHC‐II‐eF450 (eBioscience, Catalog No.48‐5321‐82), CD80‐PE (Biosciences, Catalog No.553769), CD86‐FITC (Biosciences, Catalog No.553691), F4/80‐BUV395 (Biosciences, Catalog No.565614), CD206‐PE (Biosciences, Catalog No.568273). Other reagents, including the anti‐Rat and anti‐Hamster Ig κ/negative control compensation particles set (Catalog No.552845), stain buffer (Catalog No.554656), transcription factor buffer set (Catalog No.562574), leukocyte activation cocktail with BD GolgiPlug (Catalog No.550583), and mouse BD Fc block (Catalog No.553141), were all obtained from Biosciences. All antibodies were diluted to the working concentration (0.2 µg per test). The stained cells were filtered and detected by FACS FCM (Becton Dickinson, Fortessa X20) and analyzed by Flowjo software (Tree Star, 10.6.2, Ashland, OR).

### Statistical Analyses

The quantitative data were expressed as mean ± SD. Statistical differences were calculated by GraphPad Prism software (v7.00) using unpaired Student's *t*‐test when comparing two groups. The survival curve was analyzed by the Log‐rank test. Statistical differences were recognized as *P* < 0.05 (ns, not significant, *P < 0.05, **P < 0.01, ***P < 0.001).

## Conflict of Interest

The authors declare no conflict of interest.

## Author Contributions

Y.D., J.L. and M.P contributed equally to this work. X.G., Y.D., and Y.X. designed the research. Y.D., X.G., J.L., M.P., S.L., Y.G., H.Z., Y.N., and X.Z. performed the experiments. Y.D., X.G., and T.X. analyzed the data. Y.D. and X.G. wrote the manuscript with input from all authors. X.Y. commented on the manuscript. Y.X., X.G., and T.X. supervised the experiments.

## Supporting information

Supporting Information

## Data Availability

The data that support the findings of this study are available from the corresponding author upon reasonable request.
