# Supplementary material for: Biomimetic Hydrogel‐Mediated Mechano‐Immunometabolic Therapy for Inhibition of ccRCC Recurrence After Surgery
Source: Adv Sci (Weinh). 2024 Jun 17;11(30):2308734. doi: 10.1002/advs.202308734 (PMC11321661; doi:10.1002/advs.202308734)
Supplement: Supplementary file 1 — Supporting Information [file ADVS-11-2308734-s001.pdf]

ADVANCED SCIENCE
Open Access

Supporting Information

for *Adv. Sci.*, DOI 10.1002/advs.202308734

Biomimetic Hydrogel-Mediated Mechano-Immunometabolic Therapy for Inhibition of ccRCC Recurrence After Surgery

Yunze Dong, Jun Luo, Mingliang Pei, Shuai Liu, Yuchen Gao, Hongmin Zhou, Yimingniyizi Nueraihemaiti, Xiangcheng Zhan, Tiancheng Xie, Xudong Yao, Xin Guan* and Yunfei Xu**

Supporting Information

Biomimetic Hydrogel-mediated Mechano-immunometabolic Therapy for Inhibition of ccRCC Recurrence after Surgery

Yunze Dong, Jun Luo, Mingliang Pei, Shuai Liu, Yuchen Gao, Hongmin Zhou, Yimingniyizi Nueraihemaiti, Xiangcheng Zhan, Tiancheng Xie, Xudong Yao, Xin Guan,* and Yunfei Xu**

Y. Dong, J. Luo and M. Pei contributed equally to this work.

Y. Dong, S. Liu, Y. Gao, H. Zhou, Y. Nueraihemaiti, X. Zhan, T. Xie, X. Yao, Y. Xu

Department of Urology, Shanghai Tenth People's Hospital, School of Medicine, Tongji University, Shanghai 200072, P. R. China

E-mail: 1610653@tongji.edu.cn; guan.xin@zs-hospital.sh.cn; 1300072@tongji.edu.cn

J. Luo

Department of Urology, Shanghai Fourth People's Hospital, School of Medicine, Tongji University, Shanghai 200434, P. R. China

M. Pei

Department of Orthopaedics, Shanghai Key Laboratory for Prevention and Treatment of Bone and Joint Diseases, Shanghai Institute of Traumatology and Orthopaedics, Ruijin Hospital, Shanghai Jiao Tong University School of Medicine, Shanghai 200025, China

X. Guan

Department of Ultrasound, Institute of Ultrasound in Medicine and Engineering, Zhongshan Hospital, Fudan University, Shanghai 200032, P. R. China.

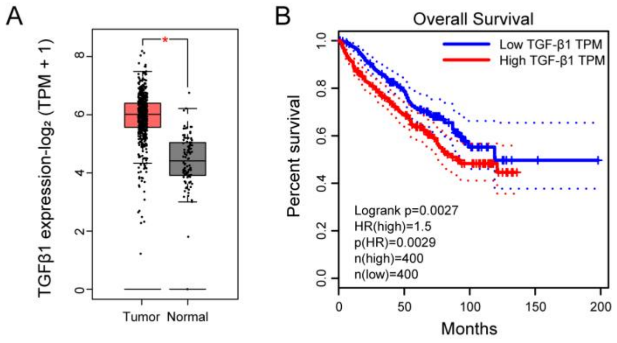

Figure S1. TGF-β1 expression in tumor tissues of ccRCC patients. A) A boxplot of TGF-β1 expression in ccRCC patients (n = 523) and healthy control (n = 100) isolated from TCGA and Genotype-Tissue Expression determined by gene expression profiling interactive analysis. The data were transformed as $\log_2$ (TPM + 1) and n is the number of biologically independent samples. B) Survival curves of ccRCC patients with high (n = 400) and low (n = 400) TGF-β1 expression. Statistical differences were calculated using a two-tailed unpaired Student's *t*-test. The survival analyses were performed by the log-rank (Mantel-Cox) test. $^*P < 0.05$.

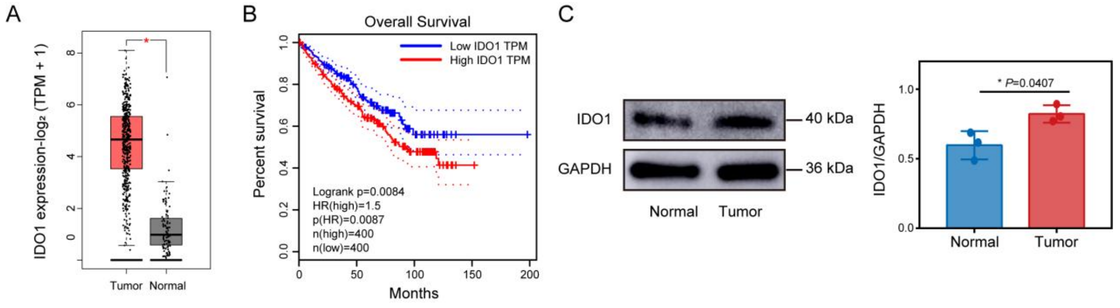

Figure S2. IDO1 expression in renal tumor tissues of ccRCC patients. A) A boxplot of IDO1 expression in ccRCC patients (n = 523) and healthy people (n = 100) isolated from TCGA and Genotype-Tissue Expression determined by gene expression profiling interactive analysis. The data were transformed as $\log_2$ (TPM + 1) and n is the number of biologically independent samples. B) Survival curves of ccRCC patients with high (n = 400) and low (n = 400) IDO1 expression. C) Western blotting and relative quantification of IDO1 protein in ccRCC patients. Data were expressed as means ± SD (n = 3). Statistical differences were calculated using a two-tailed

unpaired Student's *t*-test. The survival analyses were performed by the log-rank (Mantel-Cox) test. *$P < 0.05$.

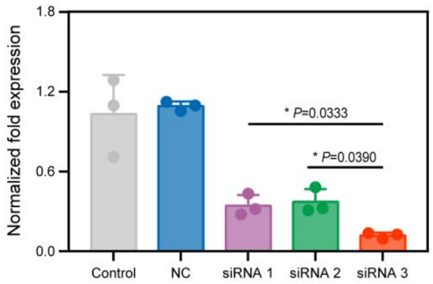

Figure S3. Gene-silencing efficiency was quantified using RT-qPCR. Among the IDO1 siRNA synthesized by the three primers provided, the one with the best silencing effect was chosen for subsequent experiment. Primers: F-5′-GTACATCACCATGGCGTATG-3′, R-5′-CGAGGAAGAAGCCCTTGTC-3′. Data were expressed as means ± SD (n = 3). Statistical differences were calculated using a two-tailed unpaired Student's *t*-test. *$P < 0.05$.

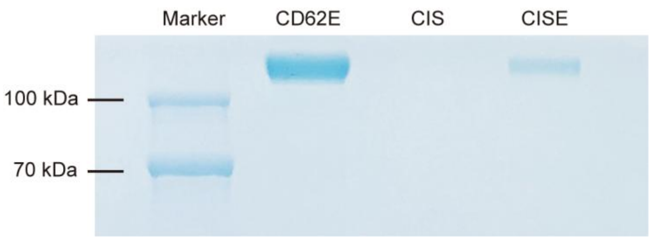

Figure S4. SDS-PAGE assay of CD62E protein in CISE NPs. SDS-PAGE protein analysis to reveal that the CD62E protein was anchored on the surface of the CISE NPs. Samples were stained with coomassie brilliant blue dye solution and visualized by a light transilluminator and a digital imaging system.

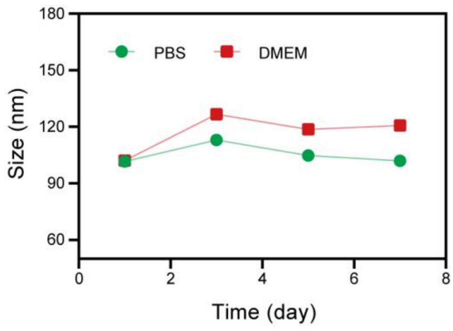

Figure S5. The size distributions of CISE NPs in PBS or DMEM for 7 days.

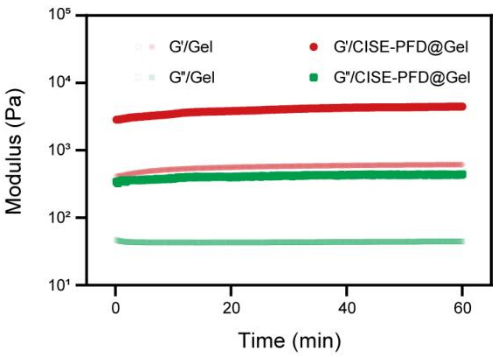

Figure S6. Rheological properties analysis of the free Gel and CISE-PFD@Gel. Variation in the moduli of the hydrogel over time. G', elastic modulus; G", viscous modulus.

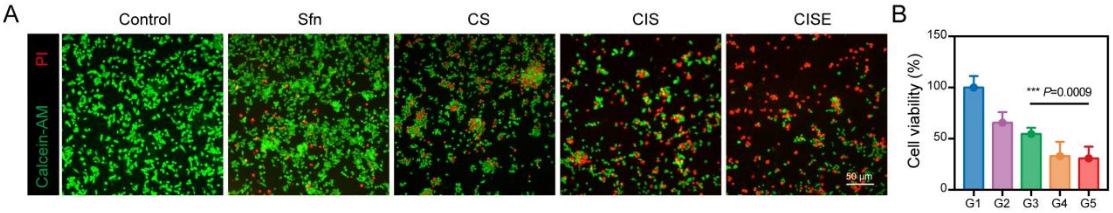

Figure S7. Cell viability assessment. A) Representative live-dead staining images of Renca cells under different culture conditions. The dead cells were stained with PI (red), while the living cells were stained with Calcein/AM (green). B) Cell viability assessed by CCK-8 kit in different groups. Data were expressed as means ± SD (n = 5). Statistical differences were calculated using a two-tailed unpaired Student's *t*-test. ****P* < 0.001. G1, control; G2, Sfn; G3, CS; G4, CIS; G5, CISE.

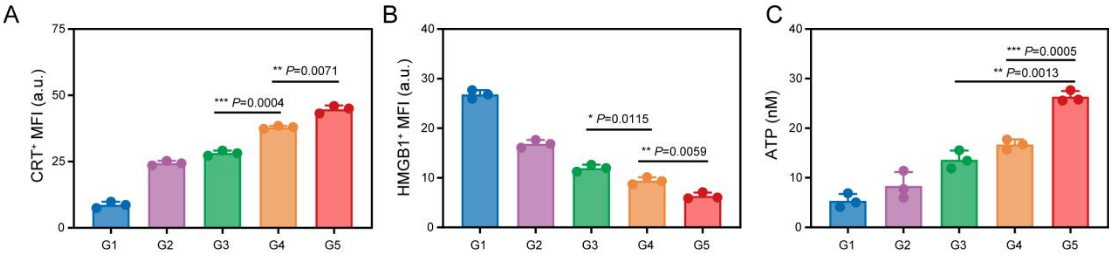

Figure S8. Quantification of the ICD effect triggered by CISE NPs. A) Corresponding mean fluorescence intensity of CRT exposure in Renca cells after

different treatments. B) Corresponding mean fluorescence intensity of HMGB1 expression in Renca cells after different treatments. C) Extracellular ATP secretion in Renca cells after various treatments. Data were expressed as means ± SD (n = 3). Statistical differences were calculated using a two-tailed unpaired Student's *t*-test. **P* < 0.05, ***P* < 0.01 and ****P* < 0.001. G1, control; G2, Sfn; G3, CS; G4, CIS; G5, CISE.

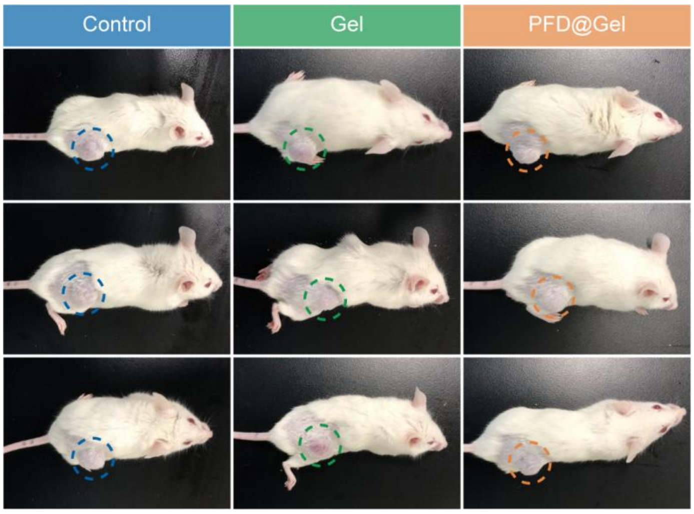

Figure S9. Representative digital photos of the treated mice from each group (Control, Gel and PFD@Gel). The circle represents the tumor site.

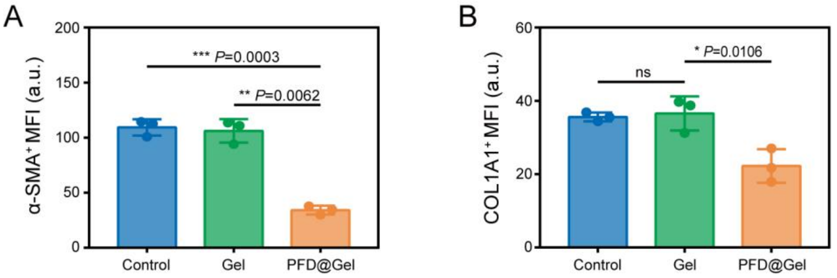

Figure S10. Quantitative analysis of α-SMA and COL1A1 in tumor tissues. A) Relative quantification of α-SMA expression within tumor sections from mice after different treatment. B) Relative quantification of COL1A1 expression within tumor sections from mice after different treatment. Data were expressed as means ± SD (n = 3). Statistical differences were calculated using a two-tailed unpaired Student's *t*-test. ns, not significant, **P* < 0.05, ***P* < 0.01 and ****P* < 0.001.

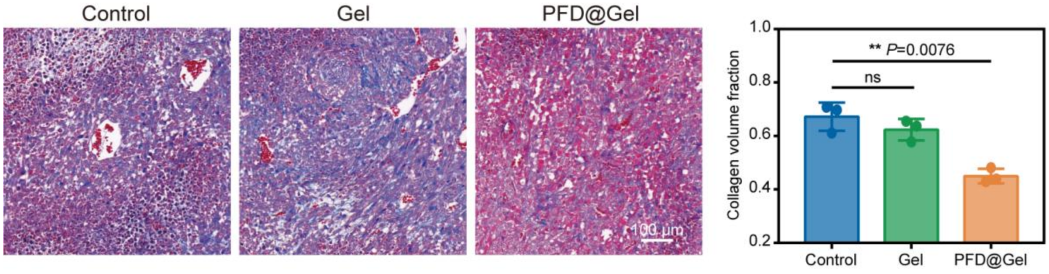

Figure S11. Masson's trichrome staining and relative quantification of tumor tissues after different treatment. Data were expressed as means ± SD (n = 3). Statistical differences were calculated using a two-tailed unpaired Student's *t*-test. ns, not significant, ***P* < 0.01.

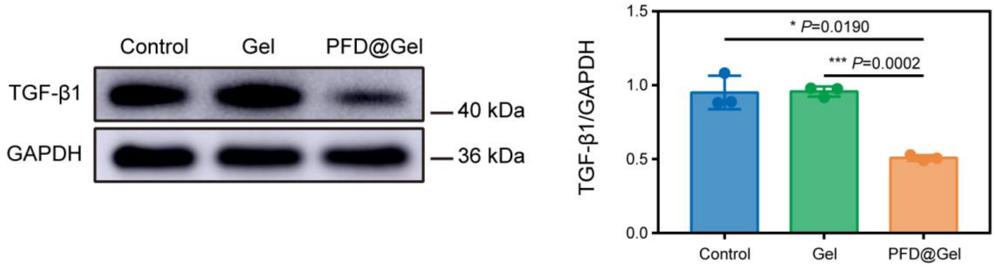

Figure S12. Western blotting assay and relative quantification of TGF-β1 expression in tumor tissues after different treatments. Data were expressed as means ± SD (n = 3). Statistical differences were calculated using a two-tailed unpaired Student's *t*-test. **P* < 0.05, ****P* < 0.001.

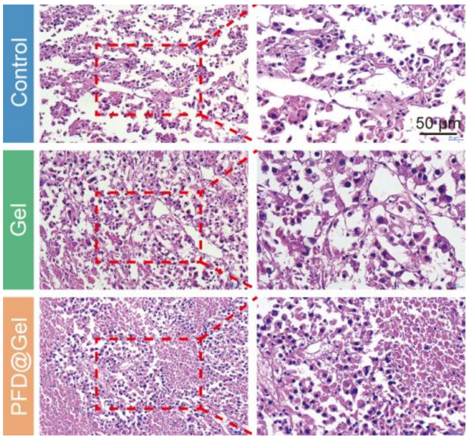

Figure S13. Representative H&E staining images of tumor tissues used for AFM test.

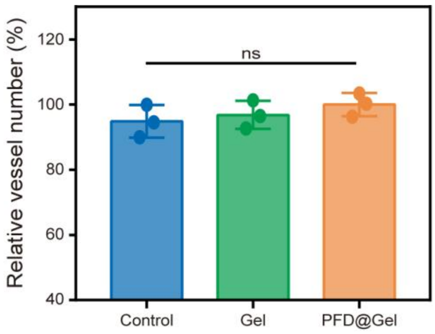

Figure S14. Relative vessel number in tumor tissues after different treatment. Data were expressed as means ± SD (n = 3). Statistical differences were calculated using a two-tailed unpaired Student's *t*-test. ns, not significant.

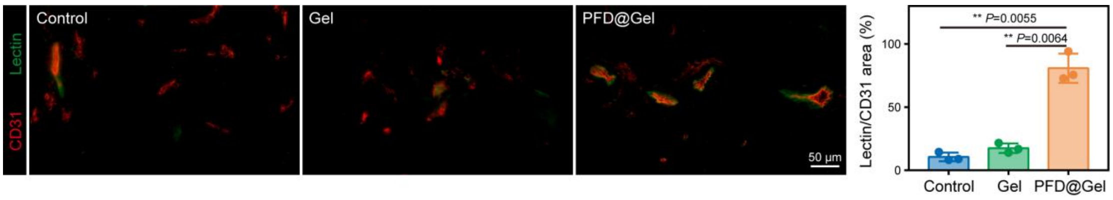

Figure S15. Immunofluorescence staining and relative quantification of tumor slices showing lectin-labeled vascular endothelium (green) and CD31-labeled total blood vessels (red). Data were expressed as means ± SD (n = 3). Statistical differences were calculated using a two-tailed unpaired Student's *t*-test. ***P* < 0.01.

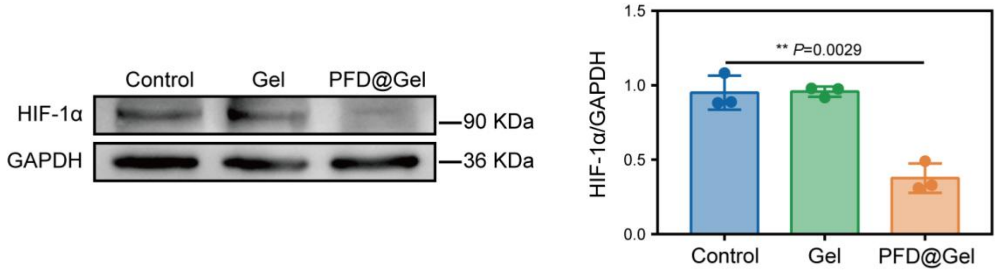

Figure S16. Western blotting strip and relative quantification of HIF-1α protein in tumors after different treatments. Data were expressed as means ± SD (n = 3). Statistical differences were calculated using a two-tailed unpaired Student's *t*-test. ***P* < 0.01.

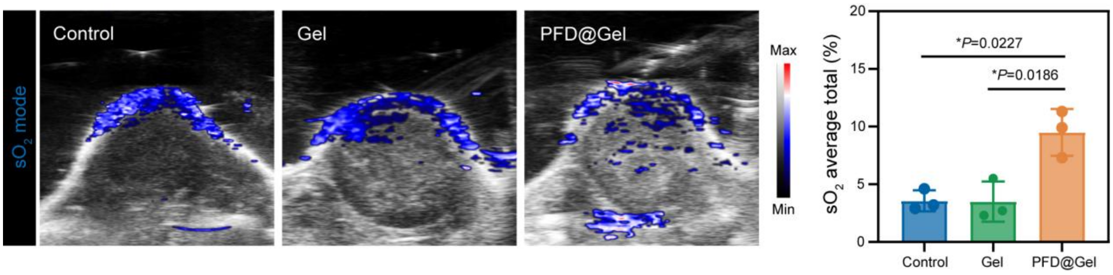

Figure S17. Photoacoustic images and corresponding quantification in oxygen saturation mode of tumors after different treatment (n = 3). Data were expressed as means ± SD (n = 3). Statistical differences were calculated using a two-tailed unpaired Student's *t*-test. *$P < 0.05$.

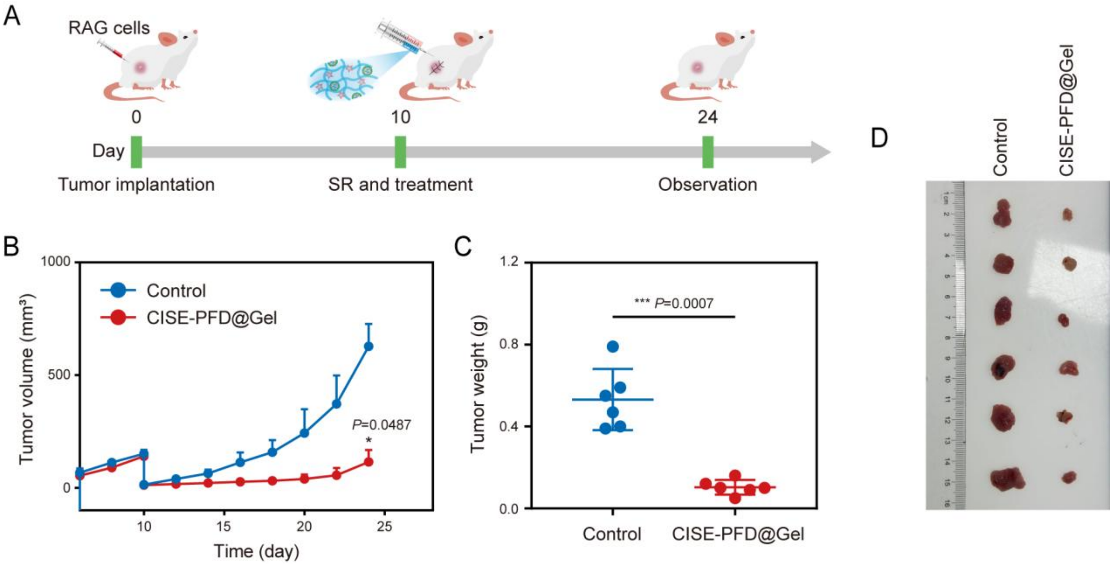

Figure S18. The CISE-PFD@Gel-augmented mechano-immunometabolic therapy against postsurgical RAG renal cell carcinoma. A) Schematic of animal experiment design. B) Residual tumor growth kinetics of mice after different treatments (n = 6). C) Weight of the excised tumors examined on day 14 after different treatments (n = 6). D) Digital photos of the excised tumors examined on day 14 after different treatments. Data were expressed as means ± SD. Statistical difference was calculated using unpaired student's *t*-test, *$P < 0.05$, ***$P < 0.001$.

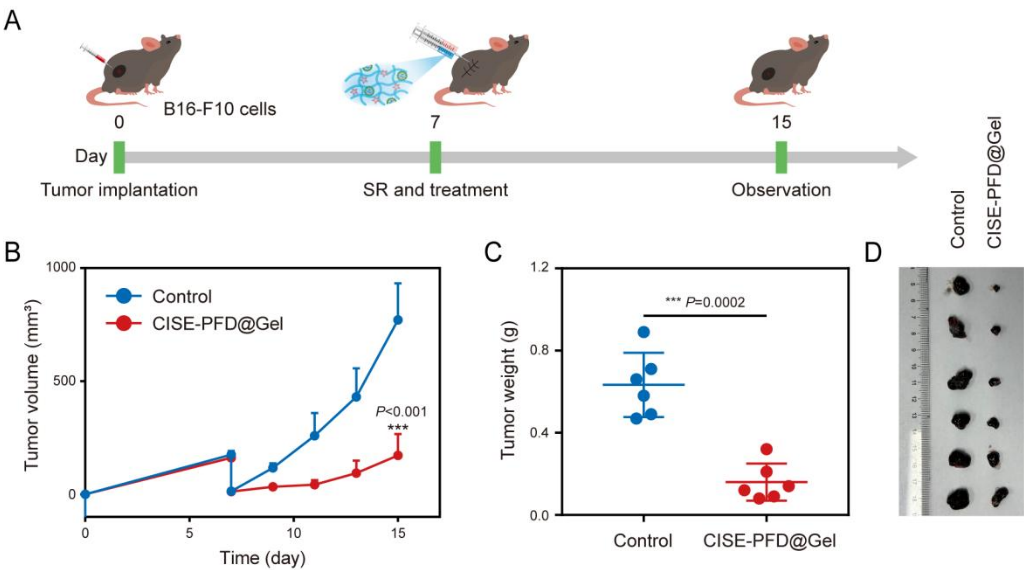

Figure S19. The CISE-PFD@Gel-augmented mechano-immunometabolic therapy against postsurgical B16-F10 melanoma. A) Schematic illustration of the study design to assess the CISE-PFD@Gel treatment efficacy in B16-F10 melanoma. B) Residual tumor growth kinetics of mice after different treatments (n = 6). C) Weight of the excised tumors examined on day 15 after different treatments (n = 6). D) Digital photos of the excised tumors examined on day 15 after different treatments. SR, surgical resection. Data were expressed as means ± SD. Statistical difference was calculated using unpaired student's *t*-test, ****P* < 0.001.

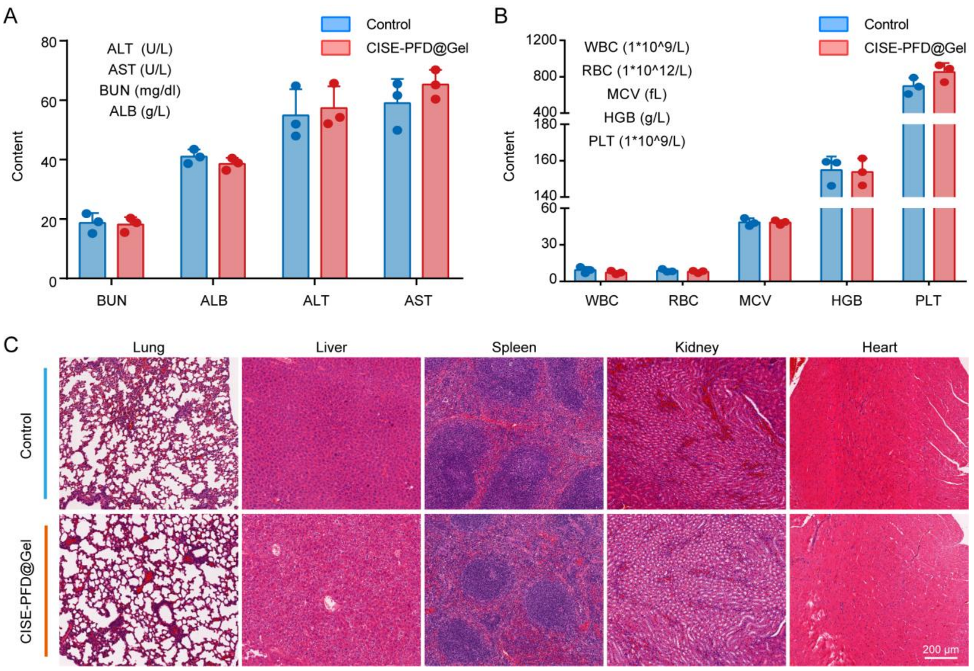

Figure S20. *In vivo* safety evaluation after CISE-PFD@Gel treatment. A) Serum biochemistry indexes including blood urea nitrogen (BUN), albumin (ALB), alanine aminotransferase (ALT) and aspartate transaminase (AST) were measured 7 days after CISE-PFD@Gel treatment. B) Blood routine indexes including leukocyte (WBC), red blood cell (RBC), mean corpuscular volume (MCV), hemoglobin (HGB), mean corpuscular hemoglobin concentration (MCHC) and platelet (PLT) were measured 7 days after CISE-PFD@Gel treatment. Data were expressed as mean ± SD (n = 3). C) Representative H&E staining of major organs from CISE-PFD@Gel-treated mice.

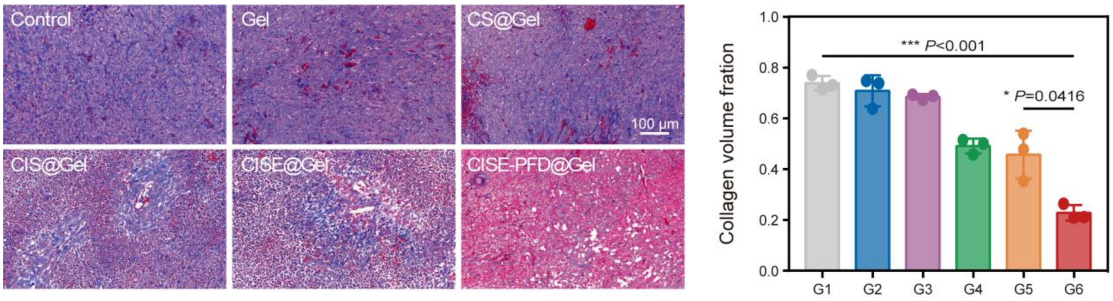

Figure S21. Masson's trichrome staining and relative quantification of tumors after different treatment. Data were expressed as means ± SD (n = 3). Statistical differences were calculated using a two-tailed unpaired Student's *t*-test. **P* < 0.05, ****P* < 0.001. G1, Control; G2, Gel; G3, CS@Gel; G4, CIS@Gel; G5, CISE@Gel; G6, CISE-PFD@Gel.

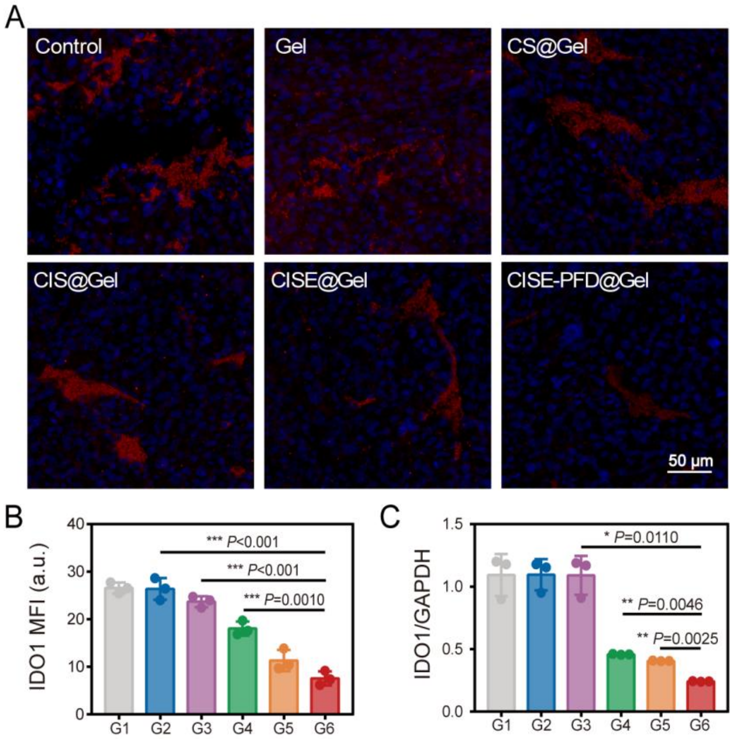

Figure S22. IDO1 silencing efficiency of CISE-PFD@Gel treatment *in vivo*. A) Representative CLSM images and B) relative quantification of IDO1 protein within tumor tissues. C) Relative quantification of IDO1 protein by western blotting within tumor tissues after different treatments. G1, Control; G2, Gel; G3, CS@Gel; G4, CIS@Gel; G5, CISE@Gel; G6, CISE-PFD@Gel. Data were expressed as means ± SD (n = 3). Statistical differences were calculated using a two-tailed unpaired Student's *t*-test. *$P < 0.05$, **$P < 0.01$ and ***$P < 0.001$.

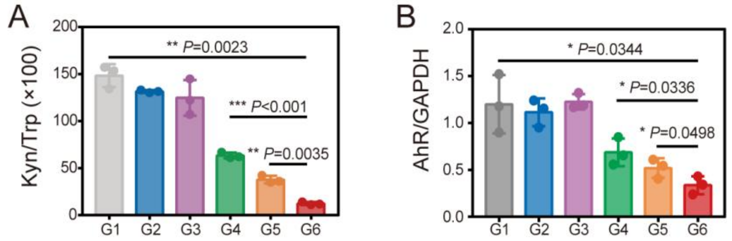

Figure S23. Regulatory effects of IDO1 downstream signaling. A) The ratio of Kyn versus Trp in tumor tissues was determined by HPLC. B) Relative quantification of AhR protein within tumor tissues after different treatments. Data were expressed as means ± SD (n = 3). Statistical differences were calculated using a two-tailed unpaired Student's *t*-test. *P < 0.05, **$P < 0.01$ and ***$P < 0.001$. G1, Control; G2, Gel; G3, CS@Gel; G4, CIS@Gel; G5, CISE@Gel; G6, CISE-PFD@Gel.

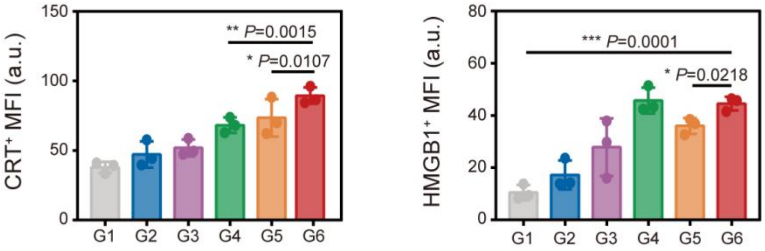

Figure S24. Relative quantification of CRT and HMGB1 proteins within tumor tissues after different treatments. Data were expressed as means ± SD (n = 3). Statistical differences were calculated using a two-tailed unpaired Student's *t*-test. **P* < 0.05, ***P* < 0.01 and ****P* < 0.001. G1, Control; G2, Gel; G3, CS@Gel; G4, CIS@Gel; G5, CISE@Gel; G6, CISE-PFD@Gel.

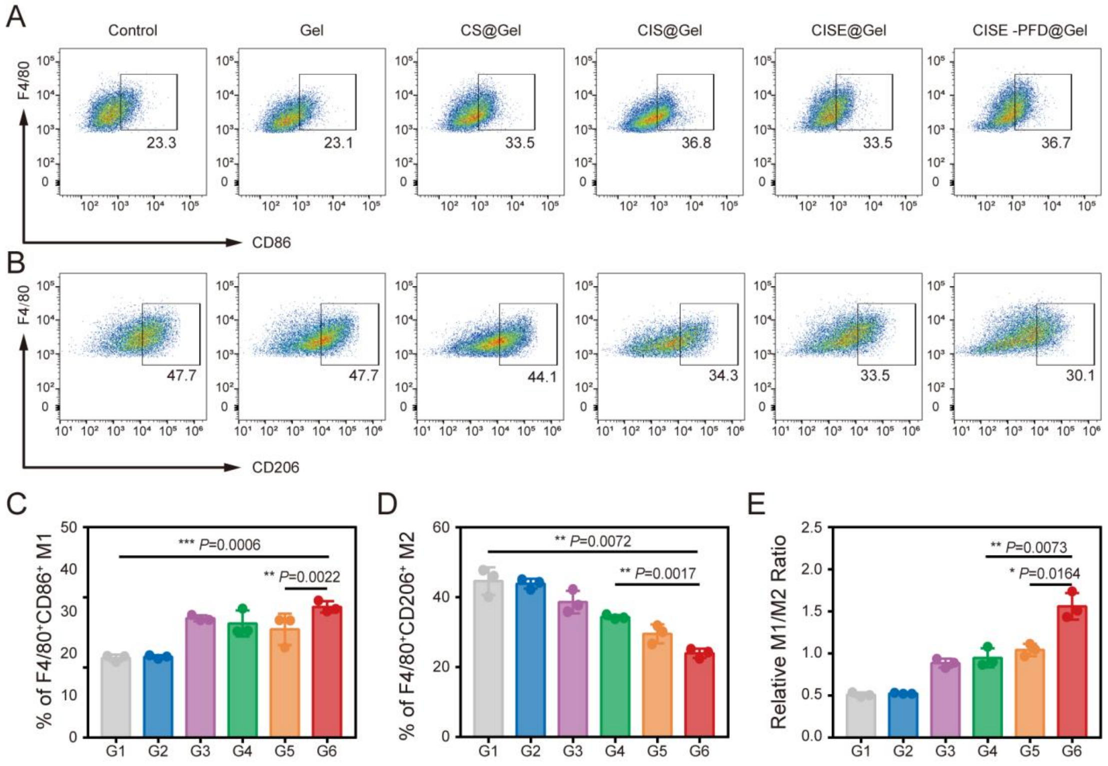

Figure S25. Infiltration of TAM subtypes within tumors. A) Representative flow cytometric images and C) relative quantification of M1-TAMs. B) Representative flow cytometric images and D) relative quantification of M2-TAMs. E) The ratio of M1-TAMs versus M2-TAMs. Data were expressed as means ± SD (n = 3). Statistical differences were calculated using a two-tailed unpaired Student's *t*-test. **P* < 0.05,

**P < 0.01 and ***P < 0.001. G1, Control; G2, Gel; G3, CS@Gel; G4, CIS@Gel; G5, CISE@Gel; G6, CISE-PFD@Gel.

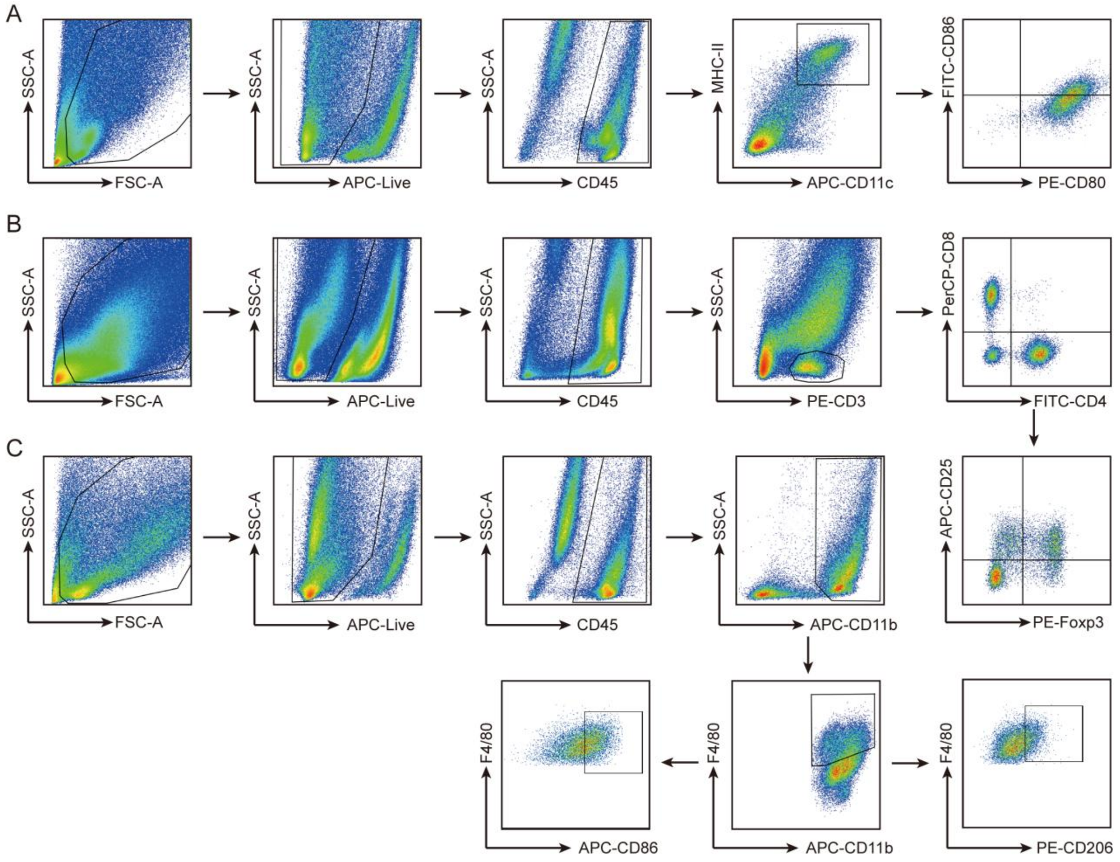

Figure S26. Gating strategy for flow cytometry analysis of immune cells. A) Gating strategy for isolating CD80^{+}CD86^{+} mDCs from tumor tissues. B) Gating strategy for isolating T cell subsets. C) Gating strategy for isolating TAMs from tumor tissues.
